# Chain hybrid feature selection algorithm based on improved Grey Wolf Optimization algorithm

**DOI:** 10.1371/journal.pone.0311602

**Published:** 2024-10-08

**Authors:** Xiaotong Bai, Yuefeng Zheng, Yang Lu, Yongtao Shi

**Affiliations:** School of Mathematics and Computer, Jilin Normal University, Siping, Jilin, China; Torrens University Australia, AUSTRALIA

## Abstract

Hybrid feature selection algorithm is a strategy that combines different feature selection methods aiming to overcome the limitations of a single feature selection method and improve the effectiveness and performance of feature selection. In this paper, we propose a new hybrid feature selection algorithm, to be named as Tandem Maximum Kendall Minimum Chi-Square and ReliefF Improved Grey Wolf Optimization algorithm (TMKMCRIGWO). The algorithm consists of two stages: First, the original features are filtered and ranked using the bivariate filter algorithm Maximum Kendall Minimum Chi-Square (MKMC) to form a subset of candidate features *S*_1_; Subsequently, *S*_1_ features are filtered and sorted to form a candidate feature subset *S*_2_ by using ReliefF in tandem, and finally *S*_2_ is used in the wrapper algorithm to select the optimal subset. In particular, the wrapper algorithm is an improved Grey Wolf Optimization (IGWO) algorithm based on random disturbance factors, while the parameters are adjusted to vary randomly to make the population variations rich in diversity. Hybrid algorithms formed by combining filter algorithms with wrapper algorithms in tandem show better performance and results than single algorithms in solving complex problems. Three sets of comparison experiments were conducted to demonstrate the superiority of this algorithm over the others. The experimental results show that the average classification accuracy of the TMKMCRIGWO algorithm is at least 0.1% higher than the other algorithms on 20 datasets, and the average value of the dimension reduction rate (DRR) reaches 24.76%. The DRR reached 41.04% for 12 low-dimensional datasets and 0.33% for 8 high-dimensional datasets. It also shows that the algorithm improves the generalization ability and performance of the model.

## 1. Introduction

Feature Selection (FS) is performed by identifying the most relevant and informative features from the original dataset and removing irrelevant and redundant features [1, 2]. It plays a crucial role in machine learning and data mining tasks [3–5]. The feature selection process aims to reduce the dimensionality of the data, improve model performance, and enhance interpret-ability. In recent years, the importance of feature selection has increased with the exponential growth of data in various domains. The main motivation behind it is to address the curse of dimensionality [6, 7], and high-dimensional data can lead to increased computational complexity, overfitting, and reduced model generalization. Feature selection not only improves the efficiency of the learning algorithm by selecting a subset of relevant features, but also helps to identify underlying patterns and relationships in the data.

Feature selection is a very essential step in machine learning that can help improve the performance and efficiency of the model. Amongst them, the three most common methods are the filter methods, the wrapper methods and the embedded methods. Filter methods focus on assessing the relevance between features and target variables through statistical measures, and common techniques include Pearson’s correlation coefficient [8], Spearman’s correlation coefficient [9], etc. Wrapper methods, on the other hand, utilize specific learning algorithms to evaluate different subsets of features to find the best combination of features. Usually heuristic algorithms [10–14] combined with classifiers [15, 16] to form wrapper algorithms are used for feature selection. Embedded methods are an approach that embeds the feature selection process into the model training. It automatically selects and adjusts the weights of the features through the model training process. Commonly, there are methods based on penalty terms [17], methods based on tree models [18], etc.

Zhou et al. [19] proposed a hybrid approach that integrates sample average approximation (SAA) and an improved multi-objective chaotic quantum-behaved particle swarm optimization (MOCQPSO) algorithm. Chance constrained programming is applied for formulating this stochastic problem. However, the computational complexity of this algorithm is high, resulting in a long running time in large-scale instances. Li et al. [20] proposed an improved binary quantum particle swarm optimization algorithm (IBQPSO). The binary PSO algorithm is a simple and efficient discrete optimization method because it guarantees a global search. And the improved algorithm solves the 0-1 knapsack problem effectively. Mean-while, a diversity maintenance mechanism is established in IBQPSO to alleviate the local optimization problem. But it lacks extensive experimental validation for different sizes and features of the knapsack problem. Gong et al. [21] aimed to solve the problems such as poor search performance of traditional particle swarm optimization algorithms in complex high-dimensional optimal problems and easy to fall into local optimum. A quantum PSO method based on diversity migration is proposed by introducing a new migration mechanism. Although subset migration strategies were adopted to enhance population diversity, they may be too conservative in exploring new search spaces. Bodha et al. [22] proposed a new quantum computing-based optimization algorithm. It is designed to solve multiple-objective mixed cost-effective emission dispatch (MEED) problem of electrical power system However, the computational overhead of the algorithm can be high, especially for large datasets. However, testing only on IEEE 14-bus and 30-bus systems failed to verify the algorithm’s performance on larger or diverse systems.

Wu et al. [23] proposed an ensemble feature selection method based on enhanced correlation matrix (ECM-EFS). The method enhances the covariance matrix, which not only considers the nonlinear relationship between different features, but also improves the interpretability and robustness of feature selection. However, the only drawback is that for high dimensional datasets, ECM-EFS may require longer computation time. Tijjani et al. [24] proposed an enhanced PSO algorithm for selecting the most informative subset of features from high dimensional data. The algorithm introduces an improved position update mechanism based on Bayesian Optimization (BO), which enhances the exploration capability of the algorithm. However, it may not deal with very high-dimensional datasets because of the high computational effort. Beheshti [25] proposed a novel fuzzy transfer function method based on Mamdani fuzzy inference system for binary particle swarm optimization. By using fuzzy transfer function, Fuzzy Binary Particle Swarm Optimization (FBPSO) enables optimal subset selection of features in the dataset with high accuracy, but the algorithm has large time cost. Li [26] proposed a binary version of the local Opposing learning Golden sine Grey Wolf Optimization algorithm (OGGWO). The algorithm enriches the population diversity and improves the convergence rate by initializing the locations of individual grey wolves using local dyadic learning maps. The golden sine algorithm is then combined with the Grey Wolf Optimization algorithm. The mean coefficient is then used to improve the autonomous search ability of individual grey wolves and avoid the algorithm from falling into a local optimum. However, the algorithm took a long time to run and could only target some specific datasets.

Although existing hybrid feature selection methods have made progress in dealing with high-dimensional datasets, they still face challenges when dealing with low-dimensional datasets. It is due to the limited number of samples in low-dimensional datasets, which may lead to unrepresentative data and susceptibility to overfitting. In this context, the selection of the most relevant and discriminative features becomes crucial to ensure that the models built are robust and well generalized. To this end, this paper focuses on the key role of feature selection and proposes a chain hybrid feature selection algorithm that explores the challenges of feature selection in the context of high and low dimensional samples. The chain hybrid feature selection algorithm refers to the use of two filter algorithms in tandem and then used in the wrapper algorithm, as if it were a chain, with one link interlocking with the other. The proposed hybrid feature selection algorithm demonstrates its favorable results on both high and low dimensional datasets through three sets of comparative experiments. The algorithm not only improves the model performance, but also reduces the runtime of the model.

The main contributions made in this paper are as follows:

1. A new filter algorithm Maximum Kendall Minimum Chi-Square (MKMC) is proposed based on the maximum relevance minimum redundancy (mRMR) criterion. Maximum Kendall is used to measure the relevance between features and labels and Minimum Chi-Square value to measure the redundancy between features.
2. Improved Grey Wolf Optimization Algorithm (IGWO): Based on the original Grey Wolf Optimization (GWO) Algorithm, a random perturbation vector is introduced to update its position. The original linearly decreasing variables are changed to random changes to avoid chance. Finally, the “Iterative oscillatory selection” method is used to replace the original random numbers in the selection of the corresponding positions.
3. Tandem filter algorithm: The original dataset is used to generate a subset of candidate features using MKMC, which is then processed by ReliefF to form a new subset of candidate features for the wrapper Algorithm.
4. In this paper, three sets of comparison experiments are used to verify that Tandem Maximum Kendall Minimum Chi-Square and ReliefF Improved Grey Wolf Optimization algorithm (TMKMCRIGWO) is superior to other algorithms. First, the IGWO is compared with other wrapper algorithms; then MKMC and ReliefF are used to combine IGWO respectively and then compared with IGWO; finally, the proposed algorithm is compared with other hybrid algorithms to prove that the proposed algorithm is superior to the comparison algorithms.

The paper is structured as follows: Section 2 describes the work related to the proposed algorithm in this paper; Section 3 describes the proposed hybrid feature selection algorithm in detail; Section 4 describes the experimental settings, and the analysis of the experimental results; and Section 5 summarizes the proposed algorithm as well as a future outlook.

## 2. Related works

This section focuses on the related contents of the proposed algorithm, A. Kendall’s correlation coefficient; B. Chi-Square Test; C. ReliefF; and D. Grey Wolf Optimization Algorithm. The algorithm of counting TMKMCRIGWO is proposed after combining and improving these contents.

### A. Kendall’s correlation coefficient

Kendall’s coefficient, which is also known as Kendall’s concordance coefficient [27], was introduced by American psychologist Maurice Kendall to measure the relevance of a relationship between two variables. The value of Kendall’s correlation coefficient, as with Pearson’s [8] and Spearman’s correlation coefficients [9], also ranges between -1 and 1. When the Kendall’s correlation coefficient is 1, it indicates that there is a positive correlation between the two variables; when the Kendall’s correlation coefficient is -1, it indicates that there is a negative correlation between the two variables; and when the Kendall’s correlation coefficient is 0, it indicates that there is no correlation between the two variables. The correlation calculation can be expressed as formula 1.

$$\begin{array}{c} \tau =\frac{n_{c}-n_{d}}{\sqrt{\left(n_{0}-n_{1}\right)\times \left(n_{0}-n_{2}\right)}} \end{array}$$ (1)

Where *n*_*c*_ denotes the number of pairs in which both variables are positively or negatively correlated at the same time; *n*_*d*_ denotes the number of pairs in which one of the two variables is positively and one is negatively correlated; *n*_0_ denotes the number of pairs in which there is no correlation between the two variables; *n*_1_ denotes the number of pairs in which one of the variables is increasing and the other is decreasing; and *n*_2_ denotes the number of pairs in which one of the variables is decreasing and the other is increasing.

Kendall’s tau is a nonparametric statistical method that is robust and widely applicable [28]. In contrast to other correlation coefficients, Kendall’s tau is unaffected by outliers and non-normally distributed data and is applicable to a wide range of data types. It can measure nonlinear relationships and is equivalent to Spearman’s correlation coefficient. In addition, it is computationally simple and efficient. Therefore, Kendall’s tau has significant advantages in analyzing data with nonlinear relationships.

### B. Chi-Square Test

Chi-Square Test [29] is a statistical method used to test for the existence of correlation between categorical variables. In chi-square test, we compare the difference between the observed frequencies and the expected frequencies to determine whether there is a significant association between the two variables. The steps of chi-square test are as follows:

1. Establish null hypothesis (*H0*) and alternative hypothesis (*H1*).

*H0*: There is no significant association between the two variables;

*H1*: There is a significant association between the two variables.

2. Calculate the Chi-square statistic according to formula 2.

$$\begin{array}{c} X^{2}=\sum \frac{{\left(O_{i}-E_{i}\right)}^{2}}{E_{i}} \end{array}$$ (2)

Where *E*_*i*_ represents the expected frequency; *O*_*i*_ is the observed frequency; and ∑ denotes accumulation over all cells.

3. Determination of degree of freedom: The degree of freedom is calculated as shown in formula 3.

$$\begin{array}{c} df=(r-1)\times (c-1) \end{array}$$ (3)

Where *r* is the number of rows and *c* is the number of columns.

4. Based on the chi-square distribution table or computer software, find the critical value at the corresponding degree of freedom and significance level.

5. Compare the calculated chi-square statistic with the critical value. If the calculated chi-square statistic is greater than the critical value, the hypothesis is rejected as there is a significant association between the two variables; otherwise, the hypothesis is accepted as there is no significant association between the two variables.

### C. ReliefF

ReliefF is a feature selection algorithm [30, 31] for selecting the most discriminating features from a dataset and. It is an instance-based feature selection method that evaluates the importance of features for a classification task by calculating the weights between them.

**Fig 1:**
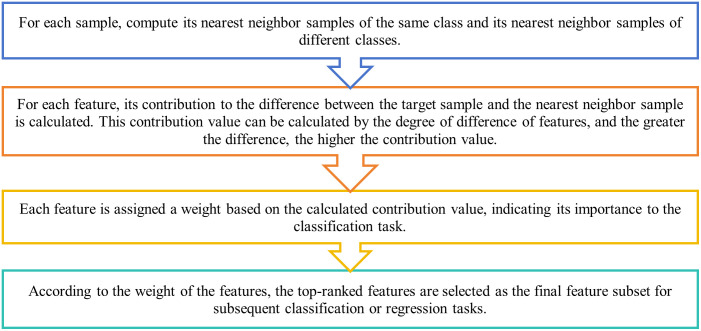
Basic steps of ReliefF. The basic steps of the ReliefF algorithm.

The ReliefF algorithm compares the feature differences between the target sample and the nearest neighbor samples to evaluate the importance of the features. The specific steps are as Fig 1:

Advantages of the ReliefF algorithm include greater robustness to noise and redundant features, and suitability for processing high-dimensional data and large-scale datasets.

### D. Grey Wolf Optimization Algorithm (GWO)

GWO [32] is an optimization algorithm based on the social behavioral characteristics of grey wolves, which was proposed by Mirjalili et al, scholars from Griffith University, Australia, in 2014. Inspired by the predatory behavior of grey wolf packs, the algorithm simulates the collaborative behavior among leaders, followers, and explorers in grey wolf packs in nature to solve optimization problems. The algorithm classifies grey wolves into four types, which are used to simulate hierarchical strata. In addition, the three main phases of finding prey, encircling prey and attacking prey were modeled. In addition, the three main phases of finding prey, encircling prey and attacking prey were simulated. Grey wolves searching for prey gradually approach the prey and encircle it, and their Mathematical model for encircling prey is given in formulas 4 and 5.

$$\begin{array}{c} D=|C\times X_{p}\left(t\right)-X\left(t\right)| \end{array}$$ (4)

$$\begin{array}{c} X\left(t+1\right)=X_{p}\left(t\right)-A\times D \end{array}$$ (5)

Where *X* denotes the position of the grey wolf; *t* is the current iteration number; *X*_*p*_ denotes the position of the prey; *D* denotes the distance between the grey wolf and the prey, which is computed in formula 5. *A* and *C* are two collaborative coefficient vectors, whose computation is shown in formulas 6 and 7.

$$\begin{array}{c} A=2a\times r_{1}-a \end{array}$$ (6)

$$\begin{array}{c} C=2\times r_{2} \end{array}$$ (7)

In formula 6, the vector *A* is used to simulate the attack behavior of the grey wolf on its prey, and its value receives the effect of *a*. *a* is the convergence factor, which is a key parameter that balances the GWO’s exploration and development capabilities. Throughout the iteration, *a* is decreased from 2 to 0. *r*_1_ and *r*_2_ are random numbers from 0 to 1.

Grey wolves can recognize the location of prey and encircling them. Grey wolves can recognize the location of potential prey (optimal solution), and when the grey wolf recognizes the location of the prey, *β* and *δ* guides the pack to encircle the prey under the leadership of *α*. The mathematical model of individual grey wolves tracking the location of prey is described as formula 8.

$$\begin{array}{c} \left\{ \begin{array}{c} D_{\alpha }=|C_{1}\times X_{\alpha }-X|\\ D_{\beta }=|C_{2}\times X_{\beta }-X|\\ D_{\delta }=|C_{3}\times X_{\delta }-X| \end{array}\right. \end{array}$$ (8)

Where *D*_*α*_ and *D*_*β*_, *D*_*δ*_ denote the distance between *α*, *β* and *δ* and other individuals, respectively. *X*_*α*_, *X*_*β*_ and *X*_*δ*_ represent the current positions of *α*, *β* and *δ*, respectively; *C*_1_, *C*_2_ and *C*_3_ are random vectors. *X* is the current position of the grey wolves, when |*A*| > 1, the grey wolves try to spread out among regions and collect prey, when |*A*| < 1, the grey wolves focus on searching for prey in a certain region or regions.

$$\begin{array}{c} \left\{ \begin{array}{c} X_{1}=X_{\alpha }-A_{1}\times D_{\alpha }\\ X_{2}=X_{\beta }-A_{2}\times D_{\beta }\\ X_{3}=X_{\delta }-A_{3}\times D_{\delta } \end{array}\right. \end{array}$$ (9)

$$\begin{array}{c} X\left(t+1\right)=\frac{X_{1}+X_{2}+X_{3}}{3} \end{array}$$ (10)

Formula 9 defines the step length and direction of an individual *ω* in a wolf pack toward *α*, *β* and *δ*, respectively, and formula 10 defines the final position of *ω*.

The GWO algorithm has a better global search ability and convergence speed, which is suitable for solving various types of optimization problems, especially in continuous optimization and multimodal optimization problems.

To enable the algorithm to jump out of the local optimum and find the global optimum, this paper proposes a chained hybrid feature selection algorithm. The first layer of dimensionality reduction is achieved by connecting the bivariate filter algorithm in tandem with the univariate filter algorithm. Then the wrapper algorithm is used to continue the exploration on the first layer of dimensionality reduction to find the optimal subset that satisfies the conditions. These works are formed based on the combination and improvement of the above, and the proposed algorithm is described in detail in the third part of this paper.

## 3. The TMKMCRIGWO algorithm

This part mainly introduces the proposed algorithm. First, the algorithm forms a subset of candidate features by tandem connection the proposed filter algorithm MKMC with the univariate filter algorithm ReliefF. Then the subset formed after the tandem filter algorithm is used in the wrapper algorithm. Finally, the optimal subset is obtained.

### A. Maximum Kendall Minimum Chi-Square (MKMC)

Based on the criterion of Maximum Relevant Minimum Redundancy (mRMR) [33], the new filter algorithm Maximum Kendall Minimum Chi-Square (MKMC) is proposed.

#### 1) Maximum Kendall (MK)

The Kendall correlation coefficients between features and labels can be calculated in the feature sets *F*_1_, *F*_2_,…,*F*_*n*_and the label *L*. While each sample contains a feature vector (*x*_1_, *x*_2_,…, *x*_*n*_) and a labeled value *y*, formula 11 is computed based on formula 1.

$$\begin{array}{c} \tau \left(F_{i},F_{j}\right)=\frac{n_{c}-n_{d}}{\sqrt{\left(n_{c}+n_{d}+n_{t}\right)\times \left(n_{c}+n_{d}+n_{u}\right)}} \end{array}$$ (11)

For two features *F*_*i*_ and *F*_*j*_, remember that the number of pairs with different ordering between the two feature sequences is *n*_*d*_, the number of pairs with the same ordering is *n*_*u*_, the number of pairs of samples that cannot be compared is *n*_*c*_, and the total number of pairs of samples is *n*_*t*_. Then, Kendall correlation coefficient of the two features *F*_*i*_ and *F*_*j*_ can be expressed as formula 11.

The maximum Kendall correlation coefficient for all features in the feature set can be expressed as formula 12.

MK(P,L)=maxp∈P(∑i=1n-1∑j=n+1nτ(Fp(i),Fp(j))(n2)) (12)

Where *P* denotes the set of feature permutations and *p* is a permutation of the feature set *P*, where *p*=1, 2, …, *n*; *τ*(*F*_*p*(*i*)_, *F*_*p*(*j*)_) is the Kendall correlation coefficient between features *F*_*i*_ and *F*_*j*_, which is computed by formula 11; (n2) is the number of combinations.

**Fig 2:**
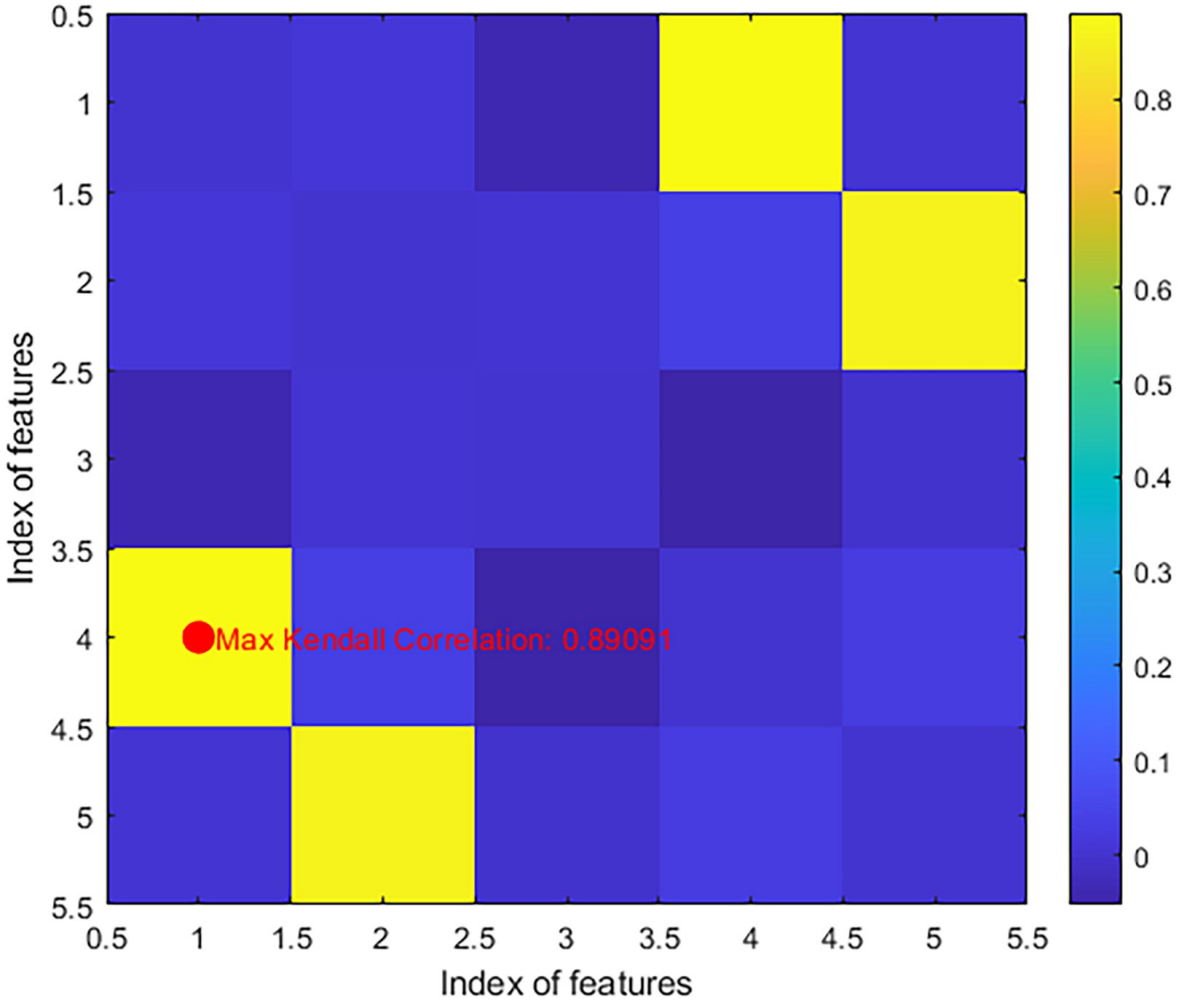
Maximum Kendall correlation coefficient. Heat map of the maximum Kendall correlation coefficient.

In this paper, we explore the relevance of different features in a prediction task using the Kendall correlation coefficient as an evaluation metric for feature selection. Suppose we take a dataset containing 100 samples and 5 features as an example. We calculated the Kendall correlation coefficient between each pair of features and labels to measure the correlation between them. And a visualization matrix was generated to show their correlation. Finally, we labeled the features that correspond to the maximum Kendall correlation coefficient to show the maximum relevance. In Fig 2, the lighter the color, the higher the relevance between the feature and the label. Its horizontal and vertical coordinates represent the indexes of the features, and we use the vertical coordinates as the label columns.

By calculating the maximum Kendall correlation coefficient, we can evaluate the maximum relevance between features and labels, helping us to select the most representative features and perform feature selection.

#### 2) Minimum Chi-Square (MC)

By the introduction of the maximum Kendall correlation coefficient, if there are still features *F*_*i*_ and *F*_*j*_, whose correlation coefficients are noted as *τ*(*F*_*i*_, *F*_*j*_), then the chi-square value is denoted as $X^{2}\left(F_{i},F_{j}\right)$.

For the association between features *F*_*i*_ and *F*_*j*_, formula 13 can be used, where *i* = 1, 2…, *n*.

$$\begin{array}{c} X^{2}\left(F_{i},F_{j}\right)=\sum \frac{{\left(O_{i}-E_{i}\right)}^{2}}{E_{i}} \end{array}$$ (13)

Where *E*_*i*_ represents the expected frequency, *O*_*i*_ is the observed frequency, and ∑ denotes accumulation over all cells.

Based on the calculation of observed frequency and expected frequency, chi-square values can be calculated to verify the degree of correlation between features.

If two different sets of features *F* and *P* are known, where the set *F* is of rows *a* and columns *b* and the set *P* is of rows *m* and columns *n*. *i*=1, 2, …, *a*, *j*=1, 2, …, *b*, *k*=1, 2, …, *m*, *l*=1, 2, …, *n*. Then it can be analyzed in four cases.

1.*X*_*ij*_: denotes the number of samples in the *ith* row and *jth* column in the set *F*, and the number of samples in the *kth* row and *lth* column that are not present in the set *P*.

2.*Y*_*ij*_: denotes the number of samples in the *ith* row and *jth* column in the set *F*, and the number of samples in the *kth* row and *lth* column in the set *P* that do not exist.

3.*Z*_*kl*_: denotes the number of samples in the *ith* row and *jth* column that do not exist in the set *F*, and the number of samples in the *kth* row and *lth* column that do not exist in the set *P*.

4.*W*_*kl*_: denotes the number of samples of the *ith* row and *jth* column that do not exist in the set *F*, and the number of samples of the *kth* row and *lth* column that do not exist in the set *P* either.

Thus, we can express the calculation of the chi-square value as formula 14.

$$\begin{array}{c} X^{2}\left(F_{i},F_{j}\right)=\frac{{\left(X_{ij}\times W_{kl}-Y_{ij}\times Z_{kl}\right)}^{2}}{\left(X_{ij}+Y_{ij}\right)\left(Z_{kl}+W_{kl}\right)\left(X_{ij}+Z_{kl}\right)\left(Y_{ij}+W_{kl}\right)} \end{array}$$ (14)

This formula can be used to calculate the chi-square value between the union of two different ranks of scale.

Therefore, the minimum chi-square value we can express as formula 15.

$$\begin{array}{c} MC\left(X^{2}\right)=min\left(X^{2}\right) \end{array}$$ (15)

**Fig 3:**
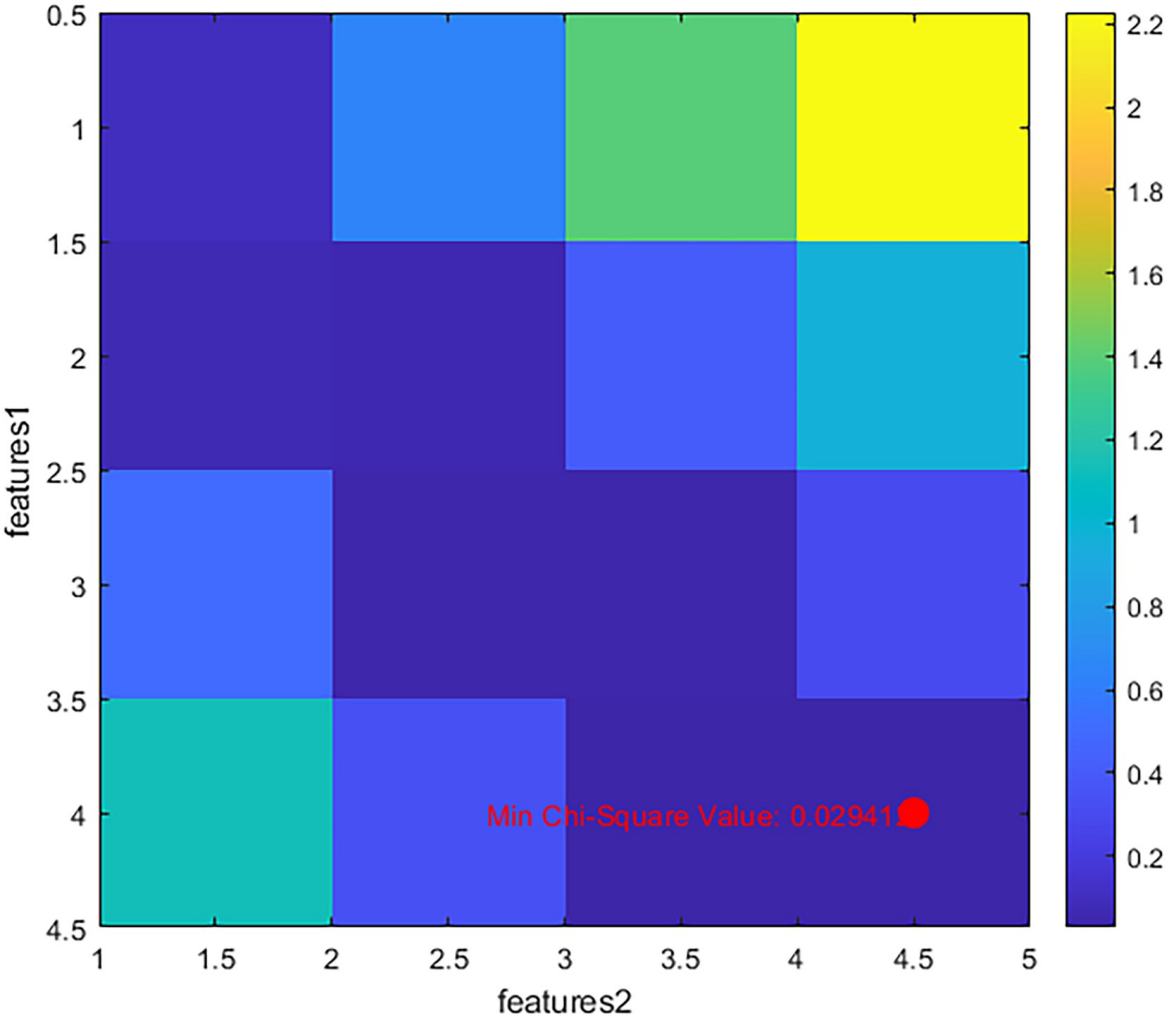
Minimum Chi-Square value. Heat map of the minimum chi-square value.

Suppose there are two existing sets, features1= {10,20,30,40} and features2= {15,25,35,45}. The two sets are calculated to obtain the chi-square value. We show the chi-square values between features and features in the form of a figure. As shown in Fig 3. The horizontal and vertical coordinates represent the index of elements in the two feature sets features1 and features2, respectively.

In Fig 3, the color shade of each cell indicates the corresponding feature-to-feature chi-square value, with darker colors indicating smaller chi-square values and lighter colors indicating larger chi-square values. Therefore, by visualizing these values using a heat map and by looking at the horizontal and vertical coordinates and the color shades of each cell, we can intuitively find the minimum chi-square value between features and features. In Fig 3 we mark the minimum chi-square value.

#### 3) Maximum Kendall Minimum Chi-Square (MKMC)

With the mRMR [29] algorithm, we can understand that in the process of feature selection, not only the relevance between features and labels, but also the redundancy between features and features should be considered. Therefore, Kendall’s correlation coefficient and chi-square test are used in this paper to measure relevance and redundancy, respectively.

Through the above two parts(Part III. The TMKMCRIGWO Algorithm, Section A. 1) Maximum Kendall(MK) and 2) Minimum Chi-Square(MC)), we can obtain formula 16, that is, we can calculate the score of each feature and output the sequence of features in descending order of the score.

$$\begin{array}{c} MKMC=max(MK\left(P,L\right)-MC\left(X^{2}\right)) \end{array}$$ (16)

Where MKMC stands for Maximum Kendall Minimum Chi-Square value. MK (*P*, *L*) and MC($X^{2}$) are obtained via formulas 12 and 15, respectively.

Feature selection was achieved by continuously selecting features with high relevance with labels and low redundancy between features. Among them, Kendall’s correlation coefficient measures the relevance between features and labels, and chi-square test measures the redundancy between features.

**Fig 4:**
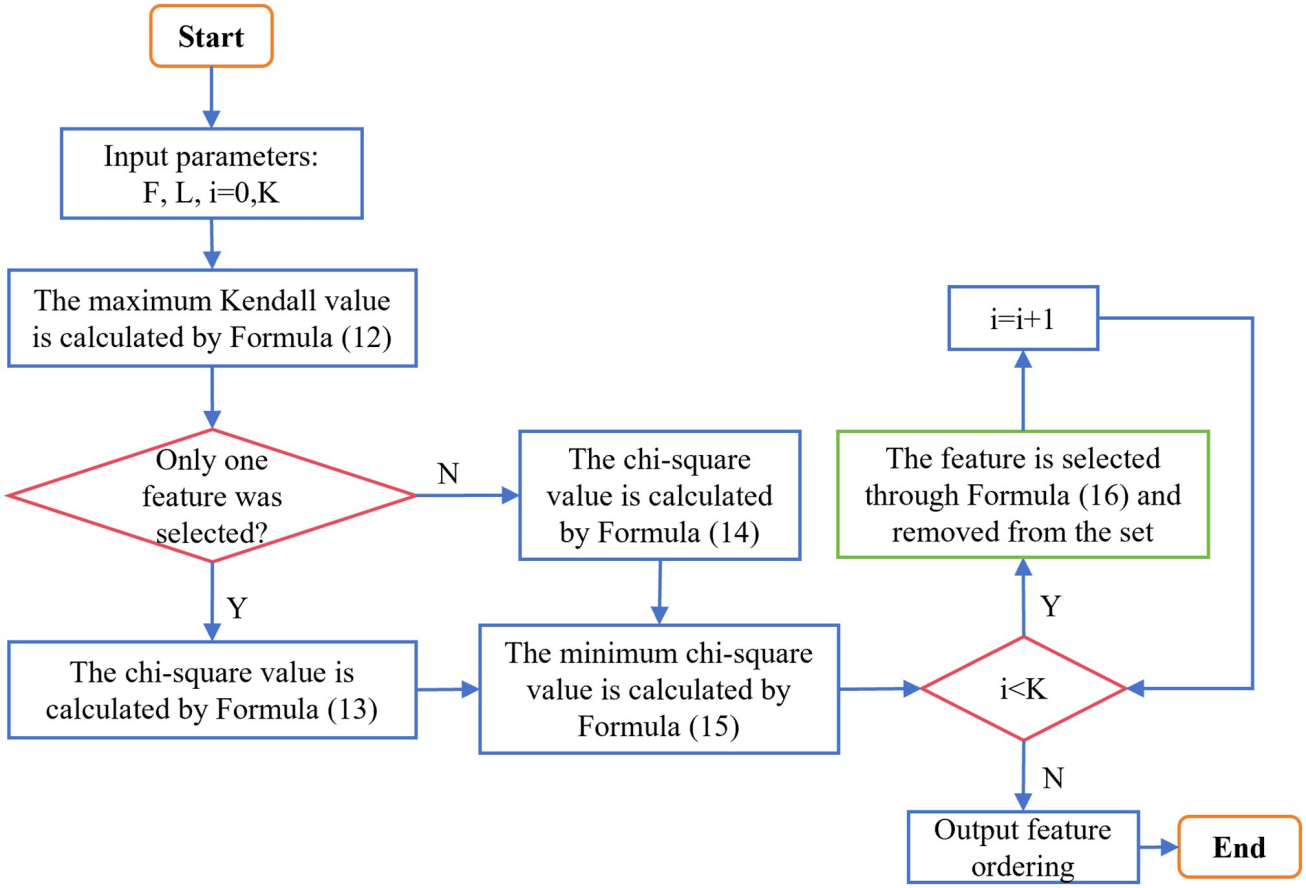
Flowchart of MKMC algorithm. This flowchart is an introduction to the proposed filter algorithm MKMC.

In Fig 4, the input parameter *F* denotes the set of all features in the dataset, and the number of features in this set is gradually reduced to empty. *L* denotes the label vector; *i* denotes the number of features selected from *F*; and *K* denotes the number of features in the dataset.

**Algorithm 1 MKMC algorithm pseudo-code**.

1 Input: *F*, *L*, *i* = 0, *K*

2 Output: Sequence of features

3 The maximum Kendall value is calculated by Formula 12

4 If only one feature is selected

5  The chi-square value is calculated by Formula 13

6 Else

7  The chi-square value is calculated by Formula 14

8 Endif

9 The minimum chi-square value is calculated by Formula 15

10 While *i* < *K*

11  The feature is selected through Formula 16 and removed from the set

12  *i* = *i* + 1

13 End While

14 Output Sequence of features

### B. Improved Grey Wolf Optimization algorithm based on random disturbance factor (IGWO)

GWO is a heuristic optimization algorithm inspired by the behavior of grey wolf packs and is commonly used to solve optimization problems. By introducing an appropriate amount of random disturbance factors, the Grey Wolf Optimization algorithm can add a certain amount of randomness in the search process, which helps to improve the global search ability and convergence speed of the algorithm.

#### 1) Position update

**Fig 5:**
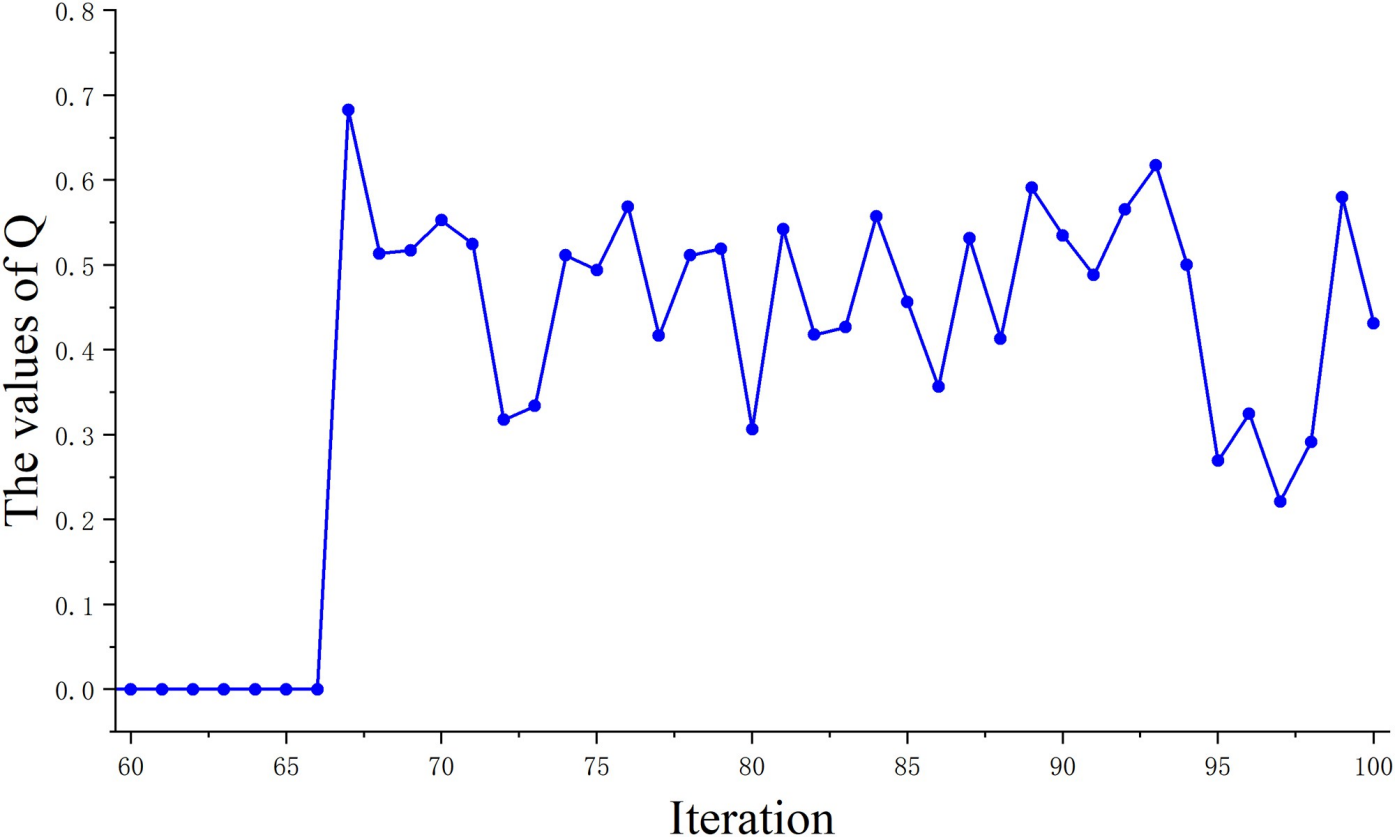
Trend of *Q* with the number of iterations. This figure illustrates the trend of *Q* when it reaches two-thirds of the maximum number of iterations.

In the traditional GWO, there are problems such as inadequate local search ability and slow convergence speed. To solve this problem, we introduce a random disturbance factor (*Q*). The search diversity of the algorithm is improved by introducing *Q* to avoid falling into local optima. Therefore, we change the position update of the traditional algorithm to formula 17, where the image of the change of *Q* with the number of iterations is shown in Fig 5.

$$\begin{array}{c} X\left(t+1\right)=Q\times \frac{X_{1}+X_{2}+X_{3}}{3} \end{array}$$ (17)

Where *Q* is the random disturbance factor which varies according to the mean and standard deviation of the position and is calculated by formula 18.

$$\begin{array}{c} Q=R\times \sigma +\bar{X} \end{array}$$ (18)

Where *R* is a random number that varies with the number of iterations; *σ* is the standard deviation of the position of each wolf in each row; $\bar{X}$ is the mean value of the position vector; *R*, *σ* and $\bar{X}$ are calculated by formulas 19–21, respectively.

$$\begin{array}{c} R=\text{sin}(rand\left(\right)\times \pi \times \frac{t}{T}) \end{array}$$ (19)

$$\begin{array}{c} \sigma =\sqrt{\frac{1}{s}\sum_{i=1}^{s}{\left(X_{i}-\bar{X}\right)}^{2}} \end{array}$$ (20)

$$\begin{array}{c} \bar{X}=\frac{1}{s}\sum_{i=1}^{s}X_{i} \end{array}$$ (21)

Where rand() is a random number from 0 to 1; *π* is the circumference, generally abbreviated as 3.14; *t* is the current iteration number; *T* is the maximum iteration number; *X*_*i*_ represents the ith position; $\bar{X}$ represents the average of all the positions; *s* is the number of positions.

In general, a smaller perturbation factor can help the algorithm avoid falling into local optimal solutions and improve the global search capability. It also does not introduce too much randomness, thus ensuring the stability and convergence of the algorithm. Therefore, in this paper, we adopt the change of introducing random perturbations at a time greater than two-thirds of the maximum number of iterations (denoted as: *T*_2/3_). At this point, the algorithm may be close to convergence, but there are still some local optimal solutions that have not been discovered. Therefore, by introducing random perturbations, it can help the algorithm to jump out of the influence of local optimal solutions and better explore the space of the global optimal solutions.

Also, introducing random disturbance factor at *T*_2/3_ helps to maintain the stability and convergence of the algorithm. Introducing randomness too early or too late may cause the algorithm to be too random or too deterministic, affecting the performance of the algorithm. Since the randomized disturbance factor is not involved in the change of each iteration, we show the change of *Q* as shown in Fig 5.

**Fig 6:**
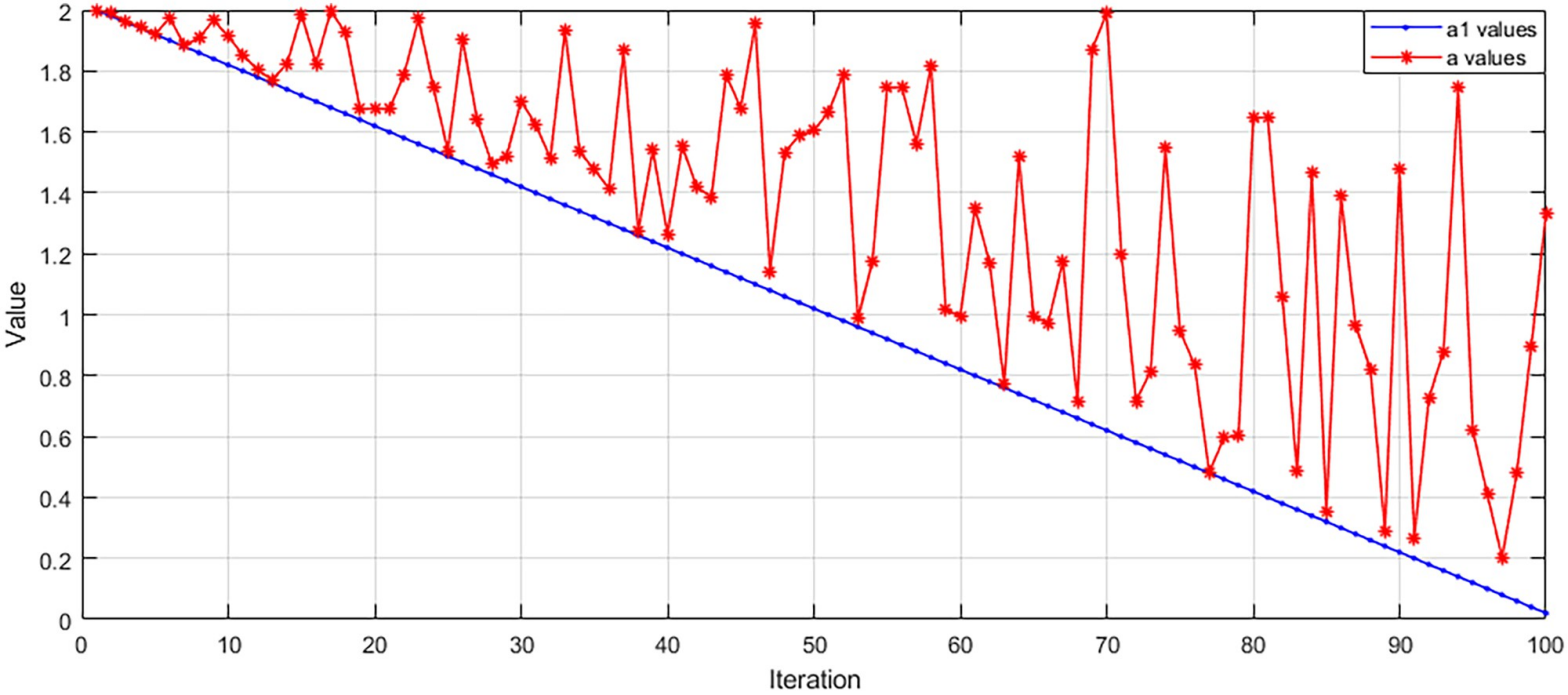
Trend of the convergence factor *a*. This figure shows the change of *a* before and after the change, where the red color represents the change after the change.

In addition, the position update is subject to changes in the collaborative vectors *A* and *C*, while *A* searches for changes in the convergence factor *a*. In the traditional method, *a* is linearly decreasing from 2 to 0. Instead, we introduce a random number to change the traditional decreasing method so that it varies randomly in the range of 0 to 2 to avoid the occurrence of chance. The variation of *a* is shown in Fig 6.

$$\begin{array}{c} a=2-rand\left(\right)\times t\times \frac{2}{T} \end{array}$$ (22)

**Fig 7:**
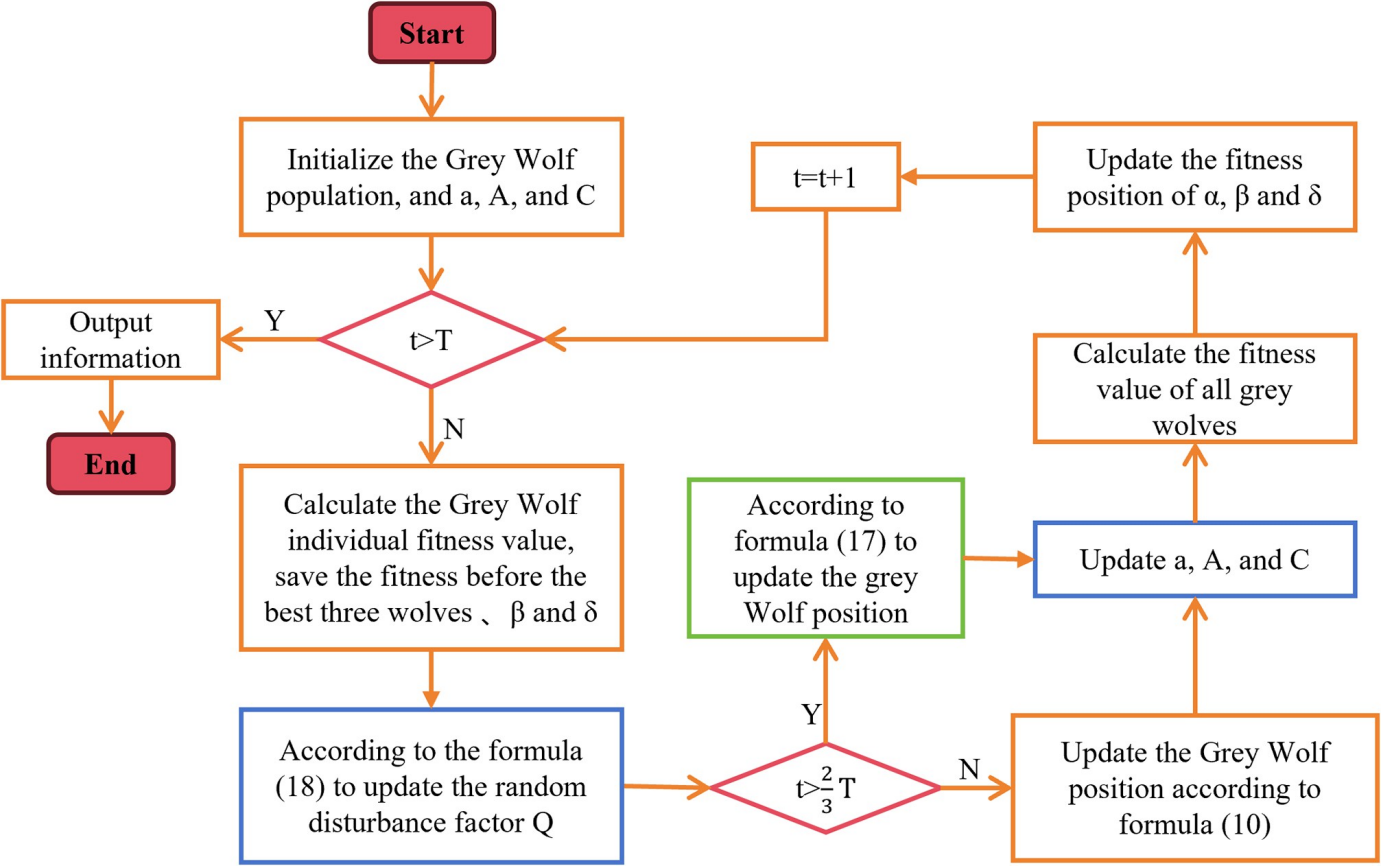
Flowchart of the improved GWO algorithm. This flowchart is an introduction to the IGWO algorithm.

The pseudo-code of the entire improved Grey Wolf Optimization algorithm is shown below, and its flowchart is shown in Fig 7.

**Algorithm 2 IGWO algorithm pseudo-code**.

1 Input: population, *a*, *A* and *C*

2 Output: Selected features

3 The fitness values of individual grey wolves are calculated according to Formula 25, and the top 3 wolves with the best fitness *α*, *β* and *δ* are preserved

4 While *t* < *T*

5  Update the random disturbance factor *Q* according to Formula 18

6  If $t>\frac{2}{3}T$

7   Update the grey wolf position according to Formula 17

8  Else

9   Update the grey wolf position according to Formula 10

10  Endif

11  Update *a*, *A*, and *C*

12  Calculate the fitness value of all grey wolves

13  Update the fitness position of *α*, *β* and *δ*

14  *t* = *t* + 1

15 End While

16 Output Selected features

In Fig 7, we represent the original GWO algorithm using orange box lines. The blue and green box lines represent the changes we made to the original GWO. In addition, we have used a red judgment box line to indicate that the changed algorithm is using a random disturbance factor to update the position at *T*_2/3_.

#### 2) Iterative oscillatory method for position selection

Positions are often chosen using a random number, the value of which is usually taken to be 0.5. Such an approach has some limitations, and there may be cases where both are greater than 0.5 or both are less than 0.5. To avoid this, we construct a parameter *r* that varies with the number of iterations and is affected by the random number. We denote it as formula 23:

$$\begin{array}{c} r=\text{sin}\left(\text{rand()}\times \pi \right)\times e^{-\frac{t}{T}} \end{array}$$ (23)

Where rand() is a random number from 0 to 1 and *e* is a natural constant.

Therefore, we define the position selection as follows:

$$\begin{array}{c} position=\{\begin{array}{cc} 1 & \text{if}\;X(t+1)>r\\ 0 & \text{otherwise} \end{array} \end{array}$$ (24)

**Fig 8:**
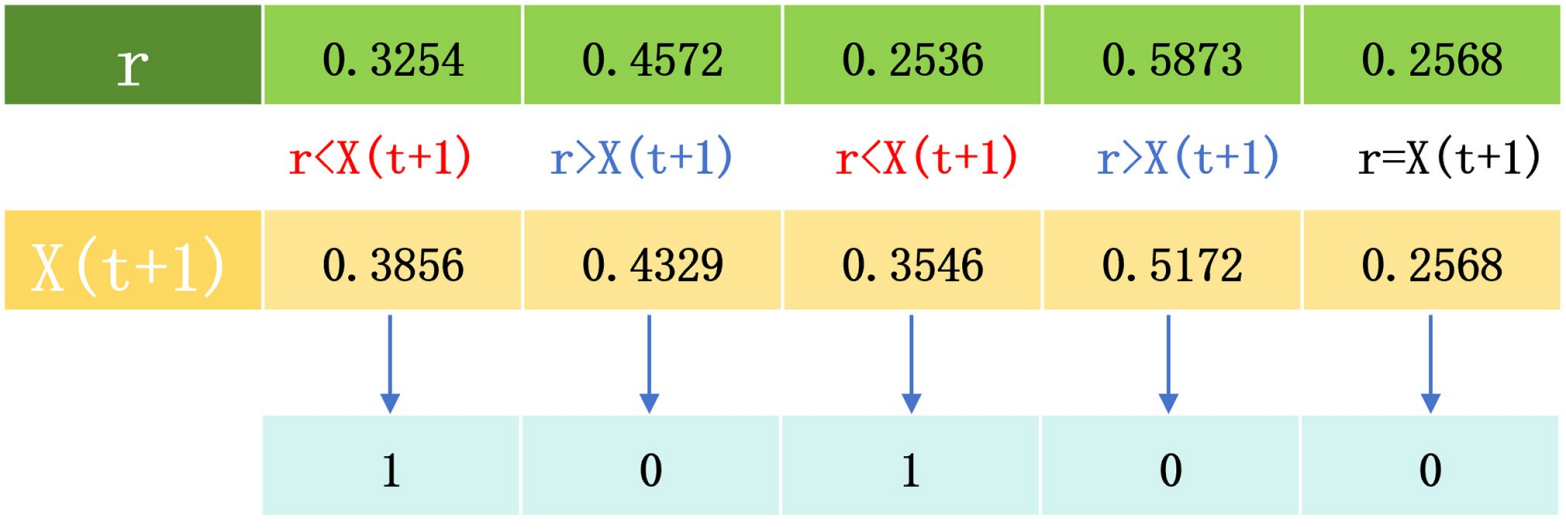
Feature correspondence position selection. This figure is an explanation of formula 24.

Where position denotes the corresponding position of the feature, if it is 1, it denotes that the position is selected; otherwise, it is not selected. As shown in Fig 8.

#### 3) Fitness function

The fitness function is a function used in evolutionary algorithms to assess the strengths and weaknesses of an individual. In the framework of evolutionary algorithms such as evolutionary strategies, the fitness of an individual is a measure of the individual’s performance and adaptability in problem solving. The fitness function is not only a criterion for judging the strengths and weaknesses of individuals, but also an important basis for evolutionary algorithms to guide the search direction in the search space.

**Fig 9:**
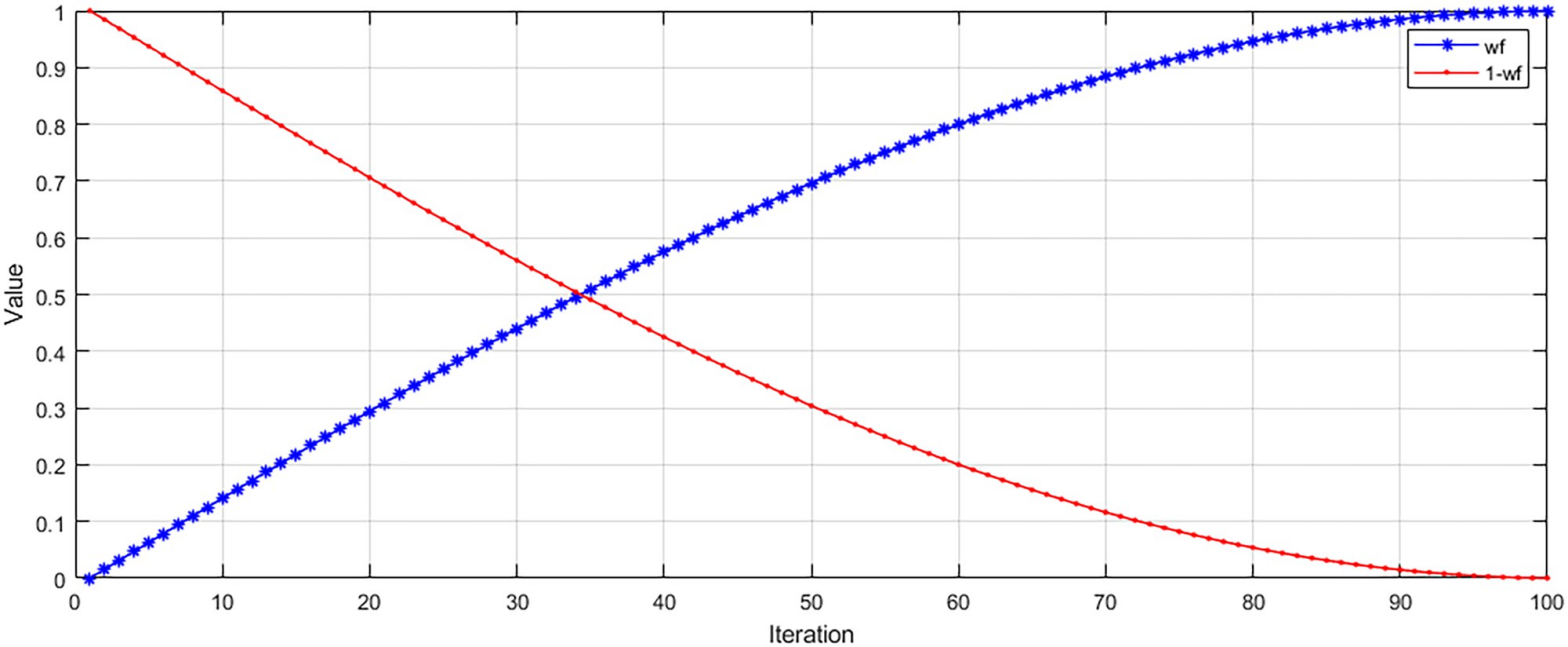
Percentage of weight value *w*_*f*_. The figure shows the trend of the proportion of ACC and LEN with the number of iterations.

However, the average classification accuracy (ACC) is usually used as the evaluation criterion of the fitness function. In this paper, we use dual metrics [34, 35], that is, the ACC and the feature subset length (LEN), to more comprehensively evaluate the merits of feature subsets. Adding the consideration of LEN to the fitness function can avoid selecting overly complex feature subsets, which reduces the model complexity and improves the generalization ability. Therefore, combined consideration of ACC and LEN can evaluate the fitness of feature subsets more effectively and thus optimize the results of feature selection. To visualize the value of the fitness function, we introduce a weight value *w*_*f*_ to regulate the proportion of ACC and LEN, as shown in formula 25. The trend of the weight value *w*_*f*_ is shown in Fig 9.

$$\begin{array}{c} fitness=w_{f}\times ACC+\left(1-w_{f}\right)\times LEN \end{array}$$ (25)

Where fitness is the fitness value; ACC and LEN are the average classification accuracy and feature subset length, respectively.

In formula 25, *w*_*f*_ is a function value that varies with the number of iterations, which we define as formula 26.

$$\begin{array}{c} w_{f}=\text{sin}\left(\frac{\pi }{2}\times \frac{t}{T}\right) \end{array}$$ (26)

Where *t* is current number of iteration and *T* is the maximum number of iterations.

### C. The proposed algorithm

This section mainly introduces the framework of the proposed algorithm (TMKMCRIGWO), including pseudo-code and flowchart. By combining the filter algorithm with the wrapper algorithm, the algorithm is avoided to fall into local optimum.

#### 1) Framework of the TMKMCRIGWO algorithm

**Fig 10:**
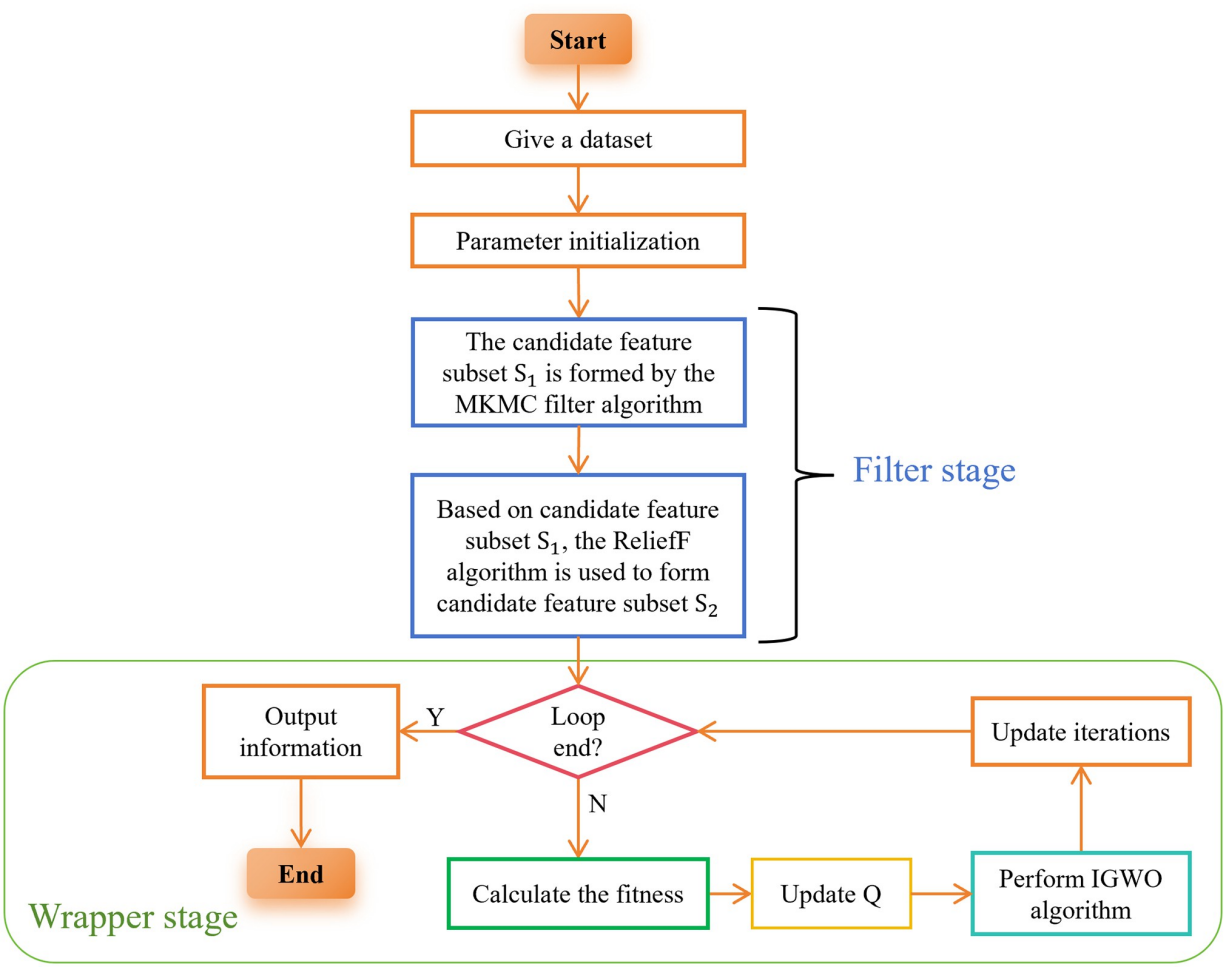
Framework of the proposed algorithm. This figure is an introduction to the proposed algorithm TMKMCRIGWO.

The key of the whole algorithm is to connect the two filter algorithms in tandem, as shown in Fig 10, firstly using the bivariate filter algorithm MKMC to generate a candidate feature subset *S*_1_. Then *S*_1_ is used as the original dataset for the ReliefF algorithm, and the candidate feature subset *S*_2_ is generated based on it, which is used in the wrapper algorithm. By connecting the two filter algorithms in tandem the features in the original dataset are incrementally downscaled to provide a better candidate feature subset for the wrapper algorithm. This method can gradually eliminate features irrelevant to the classification task in the process of feature selection, thus reducing the dimensionality of the feature space and improving the efficiency and accuracy of the algorithm.

MKMC and ReliefF algorithms can more comprehensively and accurately assess the relevance and importance between features, and then select a better-quality feature subset. Through the two-layers filter algorithms, we can get a more representative combination of features, which helps the wrapper algorithm to be more accurate and efficient in selecting the optimal subset of features.

The feature selection method using tandem filter algorithms help the proposed algorithm to jump out of the local optimum and find the global optimal solution. The final output of the algorithm is the optimal information, which helps to improve the classification accuracy and reduce the length of the feature subset, thus improving the performance and interpretability of the proposed algorithm.

#### 2) Number of selected features

The size of the dataset directly determines the speed of the algorithm, and we tend to use a fixed value for determination when dealing with large data. Such an approach has some limitations.

First, fixed values may not be able to fully consider the characteristics and complexity of the dataset, leading to inaccuracy in feature selection. Second, fixed values may not be applicable to datasets of different types or sizes and cannot flexibly respond to feature requirements in different situations. In addition, fixed values may limit the flexibility and optimization space of feature selection and fail to fully utilize the potential of feature selection. Therefore, a more intelligent and adjustable feature selection approach is needed to ensure the accuracy and efficiency of data analysis by considering the flexibility and individualized needs. Therefore, in this paper, corresponding treatments are carried out for datasets of different sizes.

In the current era of big data, data often exhibit high dimensionality [37, 38], and for high-dimensional datasets, we use a maximum-minimum determination for selection. If the dimension of the dataset is more than 1000, then, the following process is carried out, conversely, it is set to the dimension size of the dataset. The specific content is shown in formula 27.

$$\begin{array}{c} K=\text{min}(100,\text{max}\left(\left\lfloor e^{2}\right\rfloor ,\frac{\text{dim}}{3}\right)) \end{array}$$ (27)

Where *K* is the number of selected features; *e* is a natural constant; dim denotes the original dimension of the dataset; min and max denote taking the minimum and maximum values respectively. ⌊⌋ denotes rounding down.

The features we finally selected are after filter algorithm and wrapper algorithm. The first layer is to sort the features of the original dataset through the filter algorithm to form a candidate feature subject; the second layer is further dimensionality reduction based on the first layer of dimensionality reduction; the third layer uses enhancement learning algorithms, such as IGWO, based on the first two layers of dimensionality reduction, to further extract the final selected features.

**Fig 11:**
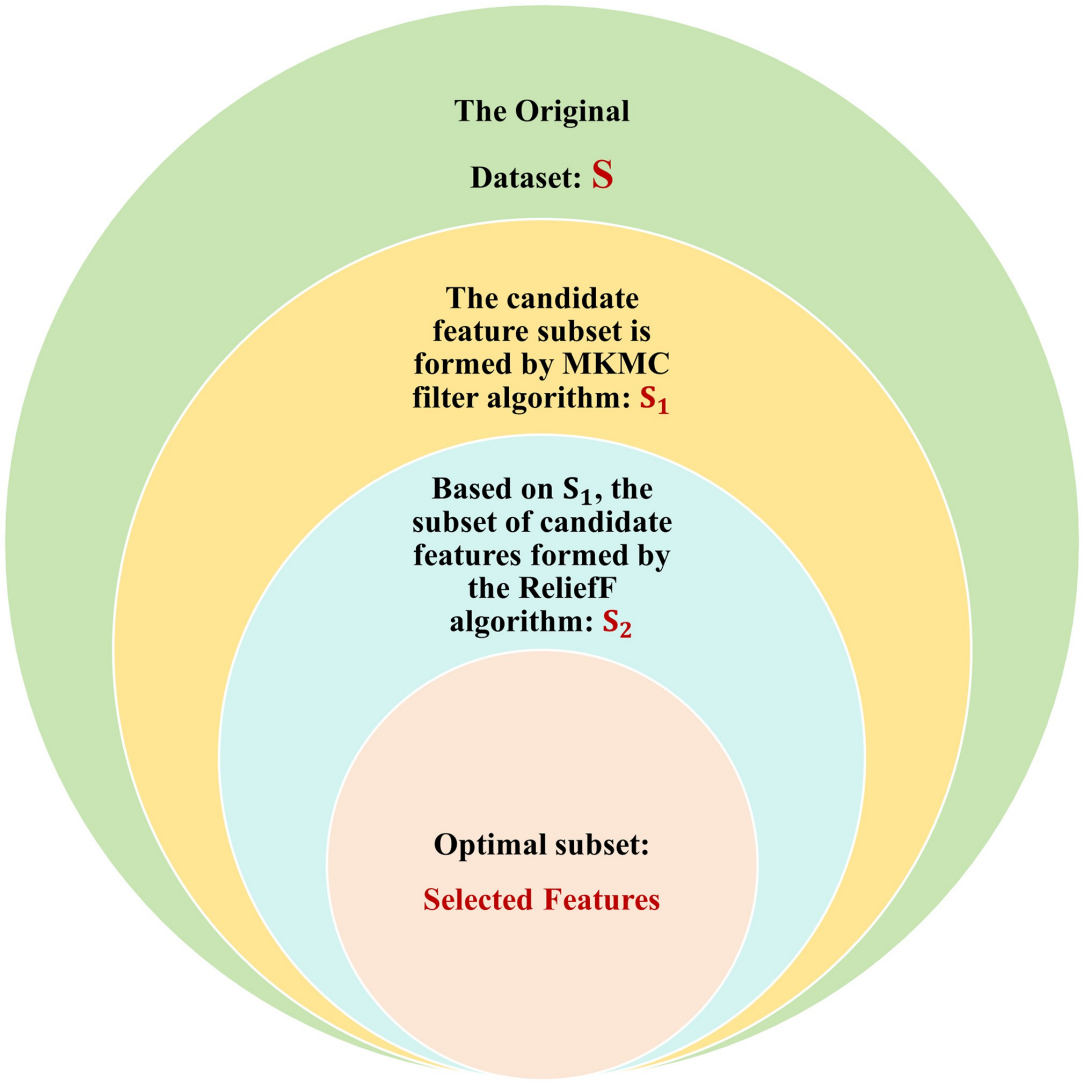
Selected features. This figure shows the process of selecting features from the original dataset.

After these three layers of dimensionality reduction, we can effectively extract the most useful features from the original data, as shown in Fig 11.

#### 3) Population initialization

For the meta-heuristic algorithm based on the GWO algorithm, the random generation of initial grey wolf positions can indeed help the population to achieve a uniform distribution in the search space. It can also increase the exploration performance of the algorithm. In the GWO algorithm, the randomness of the initial position can motivate the population to have more exploration in the initial stage. And it is more probably to traverse to the global optimal solution.

**Fig 12:**
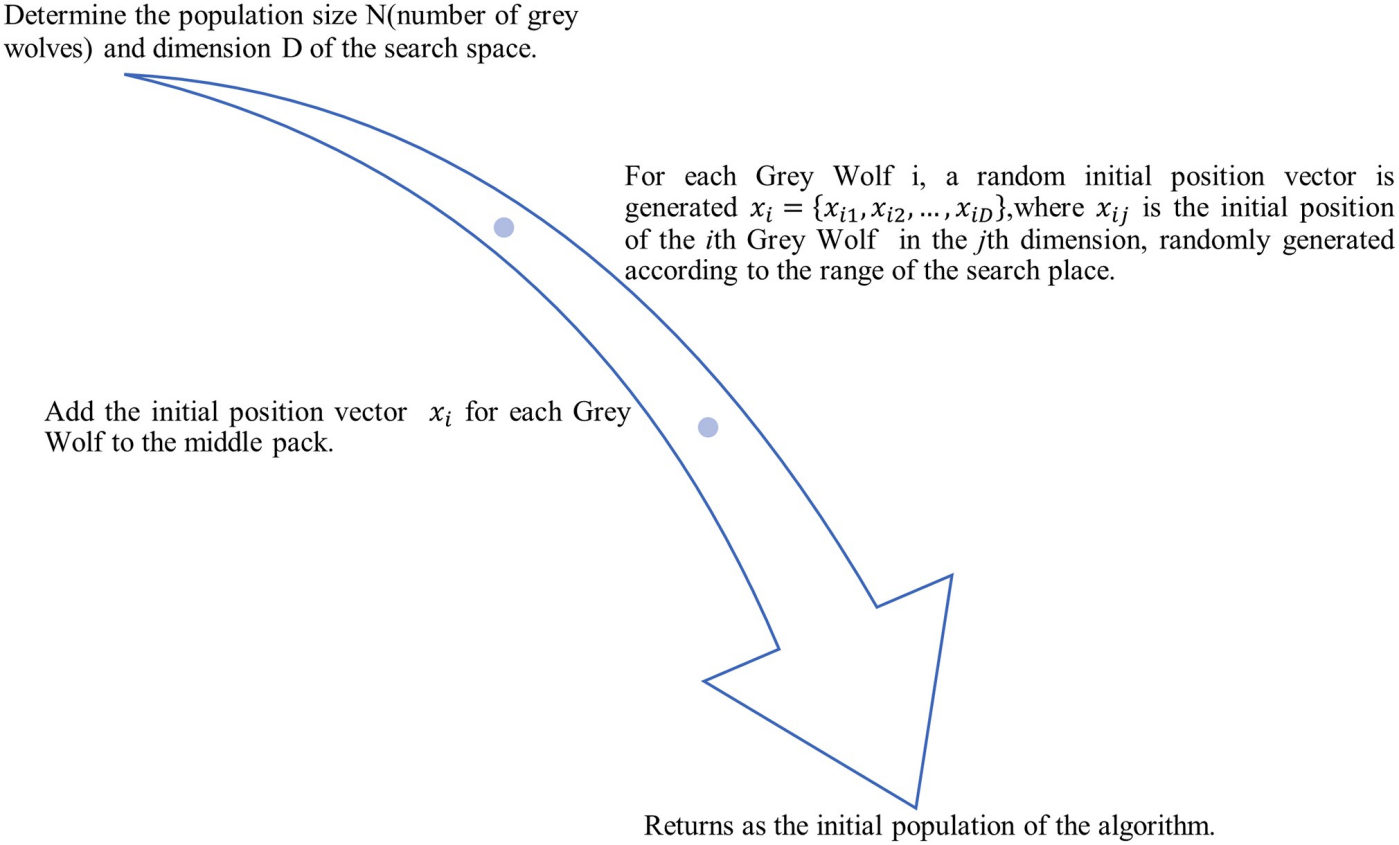
Initialization method. This figure shows the steps of population initialization by Grey Wolf Optimization algorithm.

In this paper, the initialization method [38] of randomization is used after considering the variation of the filter algorithm to rank the features. By randomly generating the initial position vectors of grey wolves, the local aggregation of the population in the search space can be effectively avoided, and a comprehensive search of the solution space is guaranteed. This uniform population distribution helps to avoid the algorithm from falling into local optimal solutions, which in turn improves the global search ability of the algorithm. The method steps of random initialization are shown through Fig 12.

#### 4) Pseudo-code and flowchart

**Fig 13:**
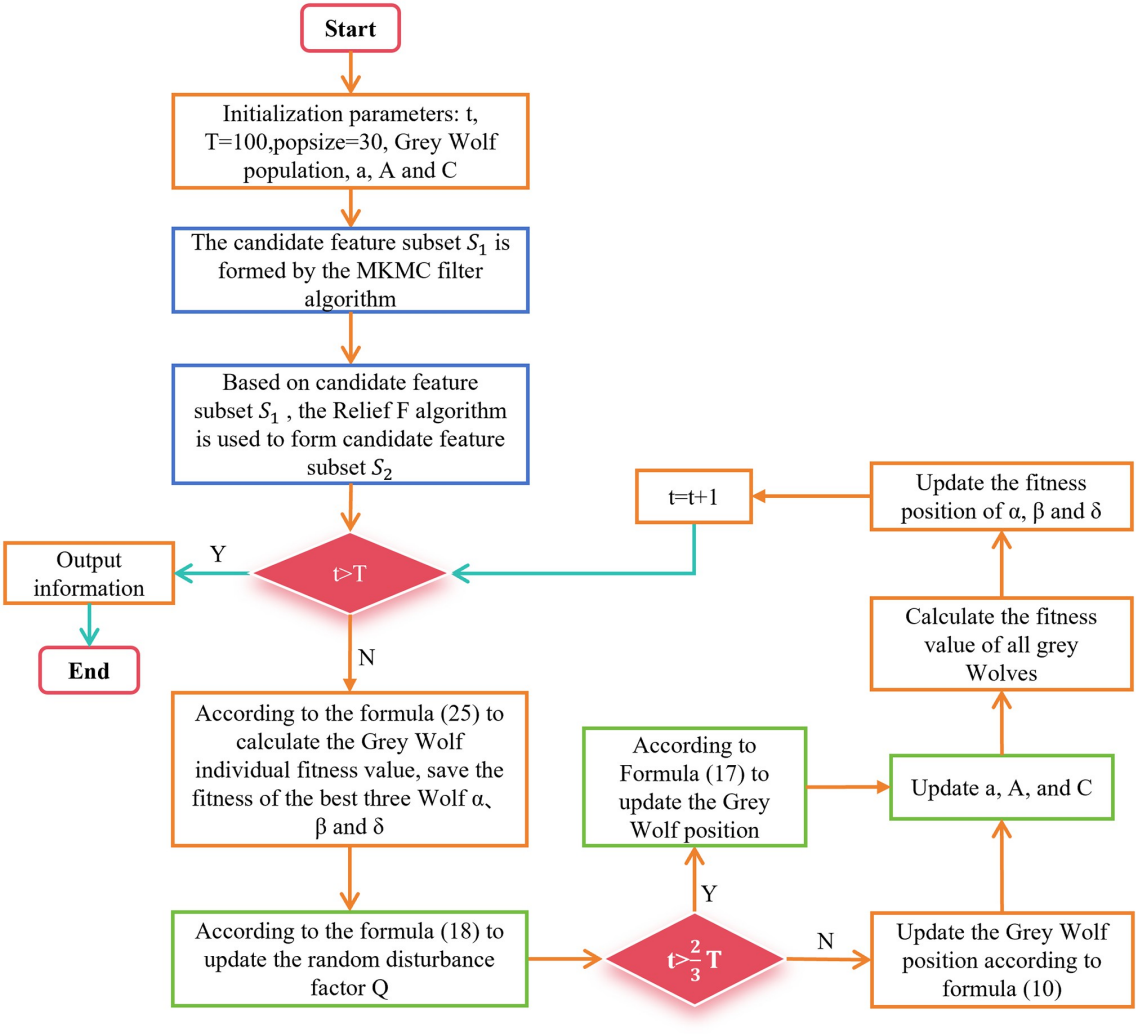
Flowchart of the TMKMCRIGWO algorithm. This flowchart is an introduction to the proposed algorithm TMKMCRIGWO.

The flowchart of the proposed algorithm TMKMCRIGWO is shown in Fig 13 and its pseudo-code is Algorithm 3.

**Algorithm 3 IGWO algorithm pseudo-code**.

1 Input: population, *a*, *A* and *C*

2 Output: Selected features

3 The candidate feature subset *S*_1_ is formed using the MKMC filter algorithm (Algorithm 1)

4 Based on *S*_1_, the ReliefF algorithm is performed to form the candidate feature subset *S*_2_

5 While *t* < *T*

6  Using Algorithm 2 IGWO

7  *t* = *t* + 1

8 End While

9 Output Selected features

## 4. Experimental results and analysis

This section introduces the dataset used for the experiments, the parameter settings of the TMKMCRIGWO algorithm and the algorithms used for the three sets of comparison experiments, and the results and analysis of the experiments.

### A. Datasets and parameter settings

#### 1) Datasets

To verify the superiority of the TMKMCRIGWO algorithm, 20 datasets were selected for a series of experiments. The details of these 20 datasets are presented through Table 1.

**Table 1:** Datasets information.

No.	Datasets	No. of Instances	No. of Features	No. of Classes	Abbreviation	Dimensionality
1	Automobile	205	25	6	Aut	Low
2	breastcancer	683	9	2	bre	Low
3	bupa liver	345	6	2	bup	Low
4	german	1000	24	2	ger	Low
5	glass	214	10	6	gla	Low
6	heart	270	13	2	hea	Low
7	ionosphere	351	34	2	ion	Low
8	Parkinsons	195	22	2	Par	Low
9	sonar	208	60	2	son	Low
10	SPECT Heart-SPECTF	267	44	2	SPE	Low
11	thyroid	215	5	3	thy	Low
12	zoo	101	17	7	zoo	Low
13	DLBCL	77	5469	2	DLB	High
14	Leukemias	72	7070	2	Les	High
15	Leukemia	72	5327	3	Lea	High
16	SRBCT	83	2308	4	SRB	High
17	Brain Tumor	50	10367	4	Bra	High
18	GLA180	180	49151	4	GLA	High
19	GLI85	85	22283	2	GLI	High
20	Leu	72	7070	2	Leu	High

Low indicates that the dataset is low-dimensional, and High indicates that the dataset is high-dimensional.

Among these 20 datasets, we selected 12 low dimensional datasets in UCI Machine Learning Repository (http://archive.ics.uci.edu/datasets), which were, Automobile, breastcancer, bupa liver, german, glass, heart, ionosphere, Parkinsons, sonar, SPECT Heart-SPECTF, thyroid, and zoo. At the same time, 8 high-dimensional datasets were selected in the Gene Expression Model Selector and Microarray Data, which were, DLBCL, Leukemias, Lukemia, SRBCT, Brain Tumor, GLA180 (https://jundongl.github.io/scikit-feature/OLD/datasets_old), GLI85, and Leu [26, 39–42]. The dimensionality of the datasets we used 2000 as a dividing line. Less than 2000 we call it a low-dimensional dataset; conversely, a high-dimensional dataset. In Table 1, we determine the dimension size of the dataset.

By conducting experiments and comparisons on these diverse datasets, we can evaluate the TMKMCRIGWO algorithm more comprehensively. The datasets in Table 1 cover the number of samples, features, and classifications of each dataset. For the convenience of experimental writing, we denote the names of the datasets in the form of abbreviations. In our experiments, we will analyze and validate these datasets using the TMKMCRIGWO algorithm. In addition, we are expected to evaluate the performance of the proposed algorithm based on the detailed information listed in Table 1 in order to demonstrate that the TMKMCRIGWO algorithm outperforms other algorithms.

#### 2) Parameter settings

Among the 9 wrapper algorithms and 7 hybrid algorithms, we all use a maximum number of iterations of 100 and a population size of 30 in order to make a fair comparison. Each dataset is tested 10 times, and the classification accuracy is the average of the 10 times. Detailed information of all algorithm parameter settings is shown in Table 2 Among the parceling algorithms, the methods for initial and actual parameters of SA, BBA, GA and CS algorithms are derived from the literature [43–46].

**Table 2:** Parameter settings.

No.	Algorithm	Parameter Settings
1	MVO+SVM	The accelerate factors (*c*_1_ and *c*_2_): 2.0, the inertia weight (*w*): 0.9
2	SA+SVM	Initial Temperature (*T*_0_):0.8, stop temperature (*T*_*f*_): 0.8^30^,cooling factor(*f*):0.8
3	BBA+SVM	loudness: 1.5, pulse rate: 0.5, minimum frequency value: 0, maximum frequency value 1
4	GA+SVM	crossover probability (*P*_*c*_): 0.7, the mutation probability (*P*_*m*_): 0.5
5	ALO+SVM	minimum of all variables(lb):0, maximum of all variables(ub):1
6	BDA+SVM	maximum weight value (*w*_*max*_):0.9, minimum weight value (*w*_*min*_):0.4
7	CS+SVM	discovery probability: 0.25, Levi’s flight parameters: 1.5
8	GWO+SVM	initial contraction coefficient: 0.5, final contraction coefficient: 0.01
9	IGWO	initial contraction coefficient: 0.5, final contraction coefficient: 0.01
10	MKMC+IGWO	initial contraction coefficient: 0.5, final contraction coefficient: 0.01
11	ReliefF+IGWO	initial contraction coefficient: 0.5, final contraction coefficient: 0.01
12	mRMR+BBA	loudness: 1.5, pulse rate: 0.5, minimum frequency value: 0, maximum frequency value 1
13	mRMR+CS	discovery probability: 0.25, Levi’s flight parameters: 1.5
14	mRMR+GWO	initial contraction coefficient: 0.5, final contraction coefficient: 0.01
15	mRMR+PSO	maximum weight value (*w*_*max*_):0.9, minimum weight value (*w*_*min*_):0.4
16	TMKMCRIGWO	initial contraction coefficient: 0.5, final contraction coefficient: 0.01

IGWO is our improved Grey Wolf Optimization algorithm and IGWO also combines with SVM.

In all these algorithms, we use Support Vector Machine as a classifier to measure the fitness function of the algorithm. The optimal feature subset is the subset of features with the highest classification accuracy based on the Support Vector Machine (SVM) classifier. Radial Basis Function (RBF) is used as the kernel function of the SVM model. And the penalty parameters and RBF parameters are selected by grid search method.

In addition, we measure the average classification accuracy by ten-fold cross-validation technique. By dividing the dataset into ten loops, each loop is divided into 10 groups, 1 group for testing and 9 groups for training. Each loop produces a classification accuracy and then the average of these accuracies and the length of the resulting feature subset are used as the result of the fitness function.

Our experiments were carried out in a CPU environment, and the data were analyzed and verified by using simulation software. In the data preprocessing section, the first step is to deal with the missing values. We downloaded the original dataset and used the average of the side-by-side samples to fill in the missing values to ensure the completeness of the data. In addition, we scrutinized the outliers in the dataset and replaced these outliers using the sample mean method to ensure the reasonableness and consistency of the data.

In our experiments, we use 20 datasets and 16 algorithms. Each algorithm is run 10 times on each dataset. Among them, all the data presented in Tables 3 to 5 represent the mean values derived from the tenfold execution of each algorithm across each dataset. The utilization of mean values not only facilitates a more accurate representation of the algorithmic performance but also mitigates the impact of outlier values. Tables 3–6 are the three sets of experiments conducted, respectively, which show the performance of the 15 algorithms and the TMKMCRIGWO algorithm in terms of the average classification accuracy and the length of the corresponding feature subset achieved on the 20 datasets. The results of the TMKMCRIGWO algorithm are verified to be superior to other algorithms by the results of different algorithms running on different datasets.

**Table 3:** Comparison of average ACC and average LEN of IGWO with other 8 wrapper algorithms.

No.	Dataset	MVO+SVM		SA+SVM		BBA+SVM		GA+SVM		ALO+SVM		BDA+SVM		CS+SVM		GWO+SVM		IGWO	
		ACC	LEN	ACC	LEN	ACC	LEN	ACC	LEN	ACC	LEN	ACC	LEN	ACC	LEN	ACC	LEN	ACC	LEN
1	Aut	58.0	16.2	72.8	13.8	70.3	12.3	66.5	13.5	67.1	14.1	83.4	15.0	66.7	25.0	86.7	10.0	**86.7**	**10.0**
2	bre	97.5	**5.0**	97.3	5.6	97.2	6.5	97.0	6.3	96.9	7.0	97.5	8.0	96.9	8.1	97.5	7.6	**97.6**	5.3
3	bup	74.5	6.0	72.9	4.0	71.8	4.5	70.8	4.8	71.1	5.2	74.8	4.0	66.7	4.9	75.4	4.0	**75.8**	**4.0**
4	ger	75.7	21.9	78.1	14.1	77.5	17.5	75.5	12.8	75.9	17.0	77.5	13.0	76.5	24.0	77.9	11.6	**77.9**	**11.6**
5	gla	26.6	4.4	97.8	5.6	97.5	5.3	97.1	4.7	97.0	5.8	98.6	4.0	91.8	10.0	99.1	4.0	**99.3**	**4.0**
6	hea	69.6	**6.0**	86.2	9.6	85.0	8.3	83.6	7.5	84.6	9.8	85.9	8.0	84.1	11.3	86.4	8.1	**86.4**	7.4
7	ion	86.2	20.7	96.9	21.0	97.0	19.5	95.8	18.3	94.0	18.8	96.6	17.0	93.6	34.0	97.6	14.1	**97.7**	**13.2**
8	Par	84.5	15.9	92.2	12.1	89.8	12.3	88.6	14.1	88.9	11.1	97.4	13.5	86.6	15.0	98.2	10.8	**98.2**	**10.1**
9	son	77.6	43.7	83.1	26.7	79.4	24.3	70.8	29.5	71.5	28.9	93.2	33.0	69.2	34.0	96.2	18.8	**96.2**	**18.7**
10	SPE	68.8	30.3	92.3	21.1	84.2	22.1	83.1	22.3	82.1	21.0	88.1	25.0	80.0	25.0	95.0	13.2	**95.8**	**12.1**
11	thy	84.4	**2.0**	96.8	4.0	95.5	4.0	93.3	4.6	96.1	4.5	98.6	4.0	96.4	4.2	98.6	4.0	**98.7**	3.4
12	zoo	74.7	6.5	95.3	7.4	94.3	7.5	94.1	9.8	93.7	8.9	96.1	5.0	91.1	15.3	95.7	5.8	**97.3**	**4.7**
13	DLB	70.3	70.3	96.3	100.0	96.8	48.1	93.7	52.2	92.6	51.8	98.1	51.8	90.5	64.8	100.0	51.3	**100.0**	**27.7**
14	Les	58.7	72.3	91.3	100.0	94.3	48.8	87.4	50.0	85.8	49.3	91.5	46.7	85.1	75.1	99.1	33.8	**99.3**	**21.0**
15	Lea	56.9	75.0	95.4	100.0	92.7	52.3	83.1	48.9	80.4	51.7	87.9	51.6	76.0	62.4	66.4	39.9	**99.5**	**25.0**
16	SRB	71.4	54.6	100.0	54.0	99.2	52.1	95.9	54.5	95.1	55.2	99.2	54.1	97.8	100.0	100.0	54.2	**100.0**	**30.9**
17	Bra	33.6	69.0	74.1	100.0	75.4	48.1	60.8	51.5	60.8	50.6	64.8	50.1	61.3	58.5	78.0	20.6	**81.4**	**19.2**
18	GLA	47.6	81.0	69.5	96.0	73.5	47.2	69.1	50.2	67.5	50.4	70.7	46.1	62.0	100.0	77.5	22.2	**77.5**	**21.7**
19	GLI	66.9	81.7	95.6	64.3	94.2	50.3	90.3	49.0	88.7	52.1	93.4	49.5	85.3	100.0	97.5	29.1	**97.7**	**19.8**
20	Leu	58.1	74.5	91.3	100.0	95.6	46.9	87.9	50.4	87.2	46.1	91.48	47.5	84.9	75.1	98.9	31.6	**98.9**	**20.2**

Bold indicates optimal values.

**Table 4:** Comparison of average ACC and average LEN of TMKMCRIGWO with IGWO, MKMC+IGWO and ReliefF+IGWO.

No.	Dataset	IGWO		MKMC+IGWO		ReliefF+IGWO		TMKMCRIGWO		LEN difference
		ACC	LEN	ACC	LEN	ACC	LEN	ACC	LEN	
1	Aut	86.7	10.0	86.9	7.9	86.8	7.9	**87.1**	**7.3**	-0.6
2	bre	97.6	5.3	97.6	5.4	97.6	5.5	**97.7**	**5.0**	-0.3
3	bup	75.8	4.0	75.4	4.0	75.6	4.0	**75.9**	**4.0**	-0.0
4	ger	77.9	11.6	77.9	11.4	78.0	12.3	**78.2**	**11.0**	-0.4
5	gla	99.3	4.0	99.3	3.8	99.4	4.0	**99.6**	**3.7**	-0.1
6	hea	86.4	7.4	86.0	5.9	86.1	7.5	**86.5**	**5.7**	-0.2
7	ion	97.7	13.2	97.7	12.3	97.6	12.3	**97.9**	**10.4**	-1.9
8	Par	98.2	10.1	98.1	8.9	98.4	10.2	**98.4**	**8.9**	-0.0
9	son	96.2	18.7	96.6	18.8	96.8	21.5	**97.0**	**18.7**	-0.0
10	SPE	95.8	12.1	95.5	12.2	96.3	11.2	**96.6**	**10.8**	-0.4
11	thy	98.7	3.4	98.7	3.3	98.6	3.4	**98.7**	**3.0**	-0.3
12	zoo	97.3	4.7	97.4	4.9	97.2	4.8	**98.0**	**4.7**	-0.0
13	DLB	100.0	28.7	100.0	40.4	100.0	42.5	**100.0**	**22.0**	-6.7
14	Les	99.3	21.0	100.0	44.4	100.0	39.6	**100.0**	**20.9**	-0.1
15	Lea	99.5	25.0	99.6	21.4	99.5	36.8	**100.0**	**18.8**	-2.6
16	SRB	100.0	30.9	100.0	27.6	100.0	51.4	**100.0**	**23.8**	-3.8
17	Bra	81.4	19.2	61.4	19.6	**92.4**	24.5	85.0	**17.3**	-1.9
18	GLA	77.5	21.7	76.4	25.9	**83.4**	24.6	78.5	**19.9**	-1.8
19	GLI	97.7	19.8	97.6	27.0	**100.0**	30.8	98.4	**18.8**	-1.0
20	Leu	98.9	20.2	100.0	39.5	100.0	39.2	**100.0**	**18.3**	-1.9

LEN difference indicates that the difference between LEN is the shortest minus the second shortest. And bold indicates optimal values, and the longest LEN is underlined.

**Table 5:** Comparison of TMKMCRIGWO with four hybrid algorithms for average ACC and average LEN.

No.	Dataset	mRMR+BBA		mRMR+CS		mRMR+GWO		mRMR+PSO		TMKMCRIGWO	
		ACC	LEN	ACC	LEN	ACC	LEN	ACC	LEN	ACC	LEN
1	Aut	86.5	14.6	85.5	14.8	87.0	10.3	74.6	11.9	**87.1**	**7.3**
2	bre	97.5	6.9	97.6	5.0	97.5	7.4	97.1	5.1	**97.7**	**5.0**
3	bup	74.9	4.2	75.2	4.0	75.7	4.0	71.7	4.0	**75.9**	**4.0**
4	ger	78.1	16.4	77.9	16.2	77.9	12.5	75.3	11.6	**78.2**	**11.0**
5	gla	99.0	5.1	98.4	3.8	99.1	4.4	98.7	4.9	**99.6**	**3.7**
6	hea	86.2	8.5	86.4	8.4	86.3	7.4	85	5.9	**86.5**	**5.7**
7	ion	97.3	16.4	97.1	19.4	97.7	12.9	96.0	13.4	**97.9**	**10.4**
8	Par	98.0	12.5	97.7	13.3	98.3	11.2	95.8	9.9	**98.4**	**8.9**
9	son	95.0	31.4	93.5	36.5	96.8	19.2	90.6	23.3	**97.0**	**18.7**
10	SPE	92.0	20.4	88.4	26.2	95.1	14.8	85.8	16.0	**96.6**	**10.8**
11	thy	98.6	3.8	98.6	3.0	98.6	4.0	98.2	3.1	**98.7**	**3.0**
12	zoo	96.6	6.5	97.2	6.6	95.9	5.9	96.2	6.9	**98.0**	**4.7**
13	DLB	100.0	52.6	100.0	43.5	100.0	51.2	100.0	34.0	**100.0**	**22.0**
14	Les	100.0	50.5	100.0	21.9	100.0	50.7	100.0	48.4	**100.0**	**20.9**
15	Lea	100.0	49.7	100.0	49.7	100.0	54.3	100.0	37.1	**100.0**	**18.8**
16	SRB	100.0	56.6	100.0	25.6	100.0	49.9	100.0	48.5	**100.0**	**23.8**
17	Bra	94.6	48.2	94.0	53.5	**96.0**	30.1	92.8	31.9	85.0	**17.3**
18	GLA	78.1	48.3	78.3	54.3	**83.7**	20.6	77.7	28.6	78.5	**19.9**
19	GLI	99.4	46.0	99.4	53.0	**100.0**	46.4	98.8	30.6	98.4	**18.8**
20	Leu	100.0	50.6	99.9	20.4	100.0	48.1	100.0	44.6	**100.0**	**18.3**

Bold indicates optimal values.

**Table 6:** Comparison of TMKMCRIGWO with four hybrid algorithms for dimension reduction rate.

No.	Dataset	mRMR+BBA	mRMR+CS	mRMR+GWO	mRMR+PSO	TMKMCRIGWO
		DRR(%)	DRR(%)	DRR(%)	DRR(%)	DRR(%)
1	Aut	58.40%	59.20%	41.20%	47.60%	**29.20%**
2	bre	76.67%	55.56%	82.22%	56.67%	**55.56%**
3	bup	70.00%	66.67%	66.67%	66.67%	**66.67%**
4	ger	68.33%	67.50%	52.08%	48.33%	**45.83%**
5	gla	51.00%	38.00%	44.00%	49.00%	**37.00%**
6	hea	65.38%	64.62%	56.92%	45.38%	**43.85%**
7	ion	48.24%	57.06%	37.94%	39.41%	**30.59%**
8	Par	56.82%	60.45%	50.91%	45.00%	**40.45%**
9	son	52.33%	60.83%	32.00%	38.83%	**31.17%**
10	SPE	46.36%	59.55%	33.64%	36.36%	**24.55%**
11	thy	76.00%	60.00%	80.00%	62.00%	**60.00%**
12	zoo	38.24%	38.82%	34.71%	40.59%	**27.65%**
13	DLB	0.96%	0.80%	0.94%	0.62%	**0.40%**
14	Les	0.71%	0.31%	0.72%	0.68%	**0.30%**
15	Lea	0.93%	0.93%	1.02%	0.70%	**0.35%**
16	SRB	2.45%	1.11%	2.16%	2.10%	**1.03%**
17	Bra	0.46%	0.52%	0.29%	0.31%	**0.17%**
18	GLA	0.10%	0.11%	0.04%	0.06%	**0.04%**
19	GLI	0.21%	0.24%	0.21%	0.14%	**0.08%**
20	Leu	0.72%	0.29%	0.68%	0.63%	**0.26%**

Bold indicates optimal values. DRR indicates Dimension Reduction Rate. And DRR is the ratio of the average LEN to the total number of features.

### B. Experimental results and comparisons

The experiments in this paper are divided into three groups, which are (1) IGWO is compared with other wrapper algorithms, (2) IGWO, MKMC+IGWO, ReliefF+IGWO and the TMKMCRIGWO algorithm are compared, and (3) the TMKMCRIGWO algorithm is compared with other hybrid algorithms.

#### 1) Experiment 1: IGWO with various types of wrapper algorithms

**Fig 14:**
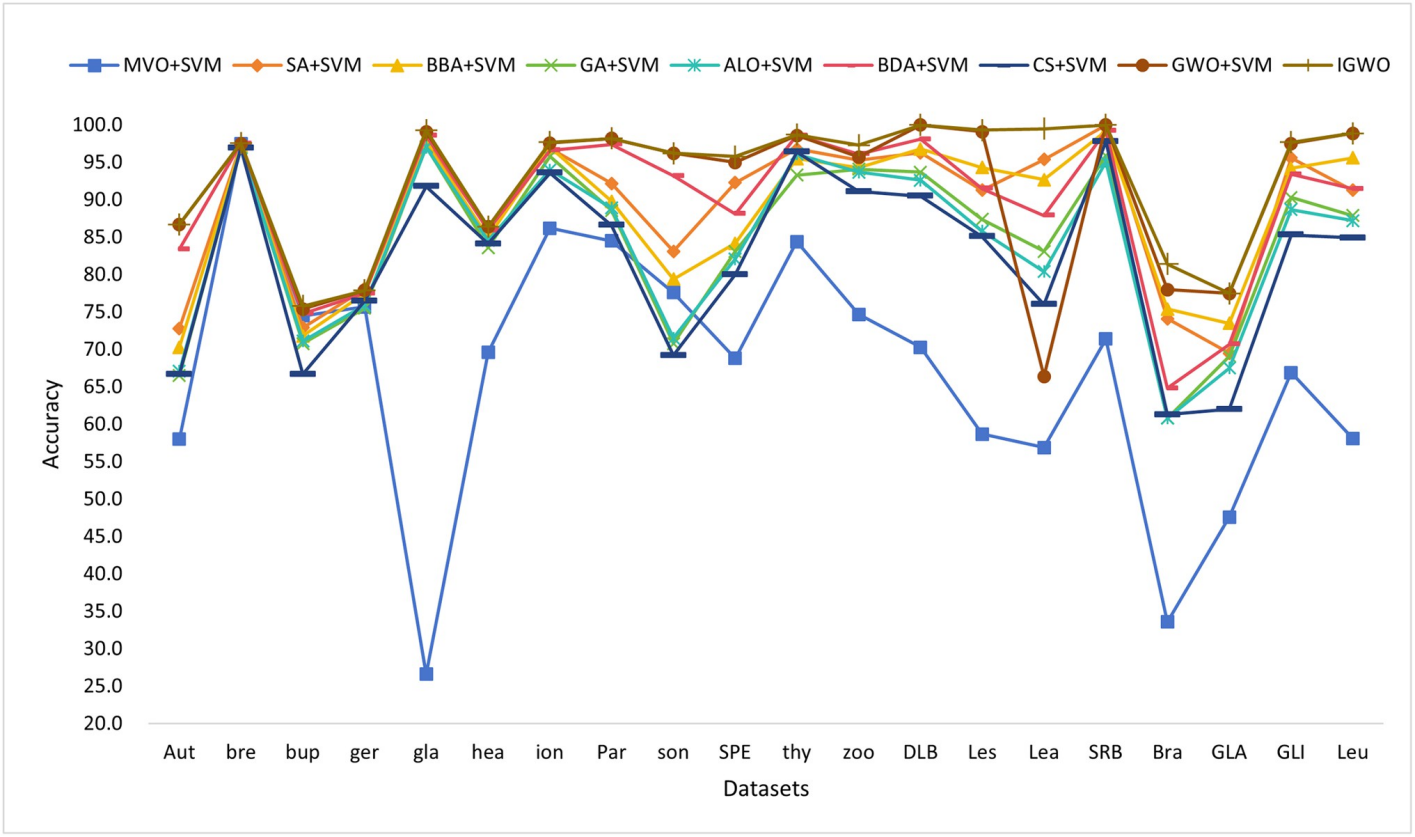
Comparison of ACC for the first set of experimental algorithms. This figure shows the ACC of the first set of experimental algorithms.

In this set of experiments, we compare the 8 wrapper algorithms with the improved Grey Wolf Optimization algorithm in this paper. With Fig 14, we can see that the average classification accuracy of IGWO is greater than the other 8 wrapper algorithms in all datasets.

Through Table 3, IGWO algorithm obtains the shortest feature subset length in most of the datasets and the average classification accuracy is much higher than other algorithms. However, on the breastcancer, heart and thyroid datasets, MVO+SVM has the shortest feature subset length among all wrapper algorithms. In the eight high-dimensional datasets such as DLBCL and Leukemia, the feature subset length of IGWO is even reduced to double digits. However, in other algorithms, the length of the feature subset is very long, even close to three digits. This also proves that the improved Grey Wolf Optimization algorithm is superior to other algorithms, which then paves the path for the second and third sets of experiments.

#### 2) Experiment 2: IGWO, MKMC+IGWO, ReliefF+IGWO and TMKMCRIGWO

In this set of experiments, IGWO is our improved wrapper algorithm. Then we combine it with the proposed filter algorithms MKMC and ReliefF algorithm respectively. Finally, we compare these three algorithms with the algorithm proposed.

On the datasets DLBCL, Leukemias, Leukemia, SRBCT, and Leu, the TMKMVRIGWO algorithm achieves an average classification accuracy (ACC) of 100%. The ACC on the sets breastcancer, glass, ionosphere, Parkinsons, sonar, SPECT Heart-SPECTF, thyroid, zoo, and GLI85 data also all reached more than 95%. And the ACC and feature subset length obtained from these datasets are better than the other three algorithms. However, on Brain Tumor, GLA180 and GLI85 datasets, the ACC obtained by ReliefF algorithm combined with the improved wrapper algorithm of in this paper is higher than that of the proposed algorithm. However, the length of the feature subset obtained by this the TMKMCRIGWO algorithm is still the smallest. We also list the specific values of feature subset lengths obtained by TMKMCRIGWO that are shorter than the second shortest feature subset length in Table 4.

#### 3) Experiment 3: TMKMCRIGWO with other hybrid algorithms

In this set of experiments, we compare the four hybrid algorithms with the TMKMCRIGWO algorithm, and it can be clearly seen that the results obtained by the TMKMCRIGWO algorithm are better than the other hybrid algorithms through 5. The ACC obtained by the TMKMVRIGWO algorithm is the highest on most of the datasets, while the LEN is the shortest in all the datasets and the value of the dimension reduction rate (DRR) is the smallest. These results show that the TMKIMCRIGWO algorithm has better performance and results in feature selection and dimensionality reduction, and has higher utility and reliability compared to other hybrid algorithms.

The ACC of the mRMR+GWO algorithm is higher than the other algorithms and the TMKMCRIGWO algorithm on the three datasets Brain Tumor, GLA180 and GLI85. However, this does not mean that it has excellent performance on all datasets. And the TMKMVRIGWO algorithm has been effective on different datasets, which shows that it has better generalization ability and stability.

Through Table 6, we can also find that in low-dimensional datasets, the DRR is generally in the range of 24% to 66.67%. In the high-dimensional dataset, the DRR is in the range of 0.04% 1.03%. It also indicates that the dimensionality reduction effect of the TMKMCRIGWO algorithm is more obvious on the high-dimensional datasets. Such experimental results show that the TMKMCRIGWO algorithm achieves better dimensionality reduction on datasets of different dimensions.

Especially on high-dimensional datasets, the TMKMCRIGWO algorithm performs more prominently compared with other algorithms and has a lower dimension reduction rate, indicating that the TMKMCRIGWO algorithm has better adaptability and effectiveness. In addition, the superiority and effectiveness of the TMKMCRIGWO algorithm is further verified by comparison experiments with other algorithms. These experimental results further validate the superiority of the IGWO algorithm and the TMKMCRIGWO algorithm in feature selection and dimensionality reduction tasks. In the first set of experiments, the IGWO algorithm performs well and outperforms the other wrapper algorithms, indicating that it has better performance and results in the feature selection phase. And in the second and third set of experiments, the proposed algorithm performs better compared to other hybrid algorithms, indicating that the TMKMCRIGWO algorithm has higher ACC in the process of dimensionality reduction.

### C. Experimental analysis

#### 1) Dimension Reduction Rate (DRR)

In this paper, the DRR refers to the ratio of the number of features we finally get to the number of features in the original dataset, the smaller the ratio, the greater the degree of dimensionality reduction of the algorithm is proved to be; on the contrary, the smaller the degree of dimensionality reduction is.

**Fig 15:**
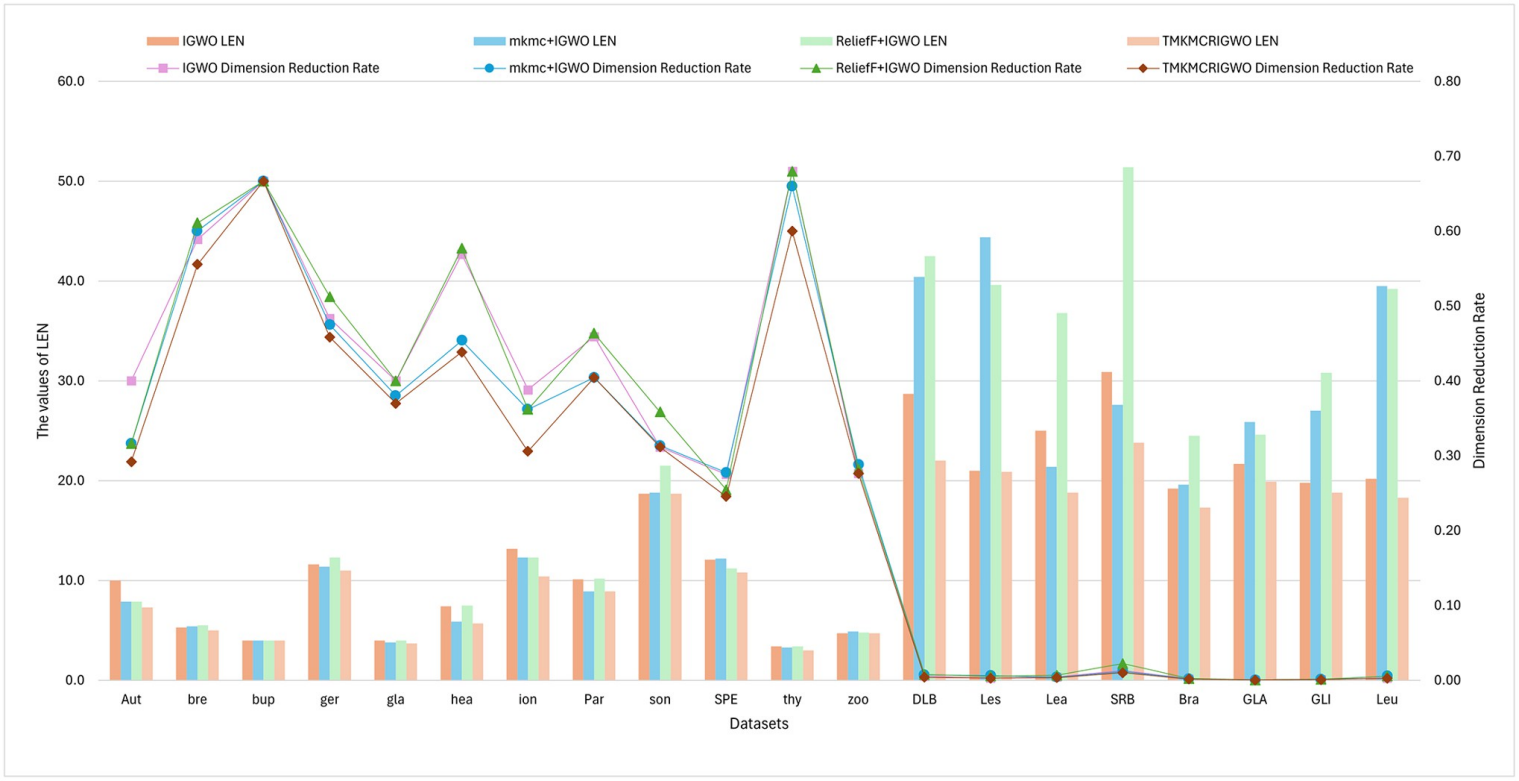
LEN and dimension reduction rates for the second set of experiments. This figure shows LEN and DRR for the second set of experimental algorithms on 20 datasets.

In Fig 15, we compared the results of the second set of experiments in this paper. The feature subset length and the dimensionality reduction rate of each dataset are shown in the form of bar charts and line figures. The feature subset length of the TMKMCRIGWO algorithm is lower than the other three algorithms and the DRR is also the lowest on all datasets. It indicates that the TMKMCRIGWO algorithm is superior to the other algorithms. It further proves that the DRR of the TMKMCRIGWO algorithm is better than the other algorithms, no matter it is a high-dimensional dataset or a low-dimensional dataset. Meanwhile, it indirectly demonstrates that the TMKMCRIGWO algorithm has a certain applicability to some high-dimensional and low-dimensional datasets.

**Fig 16:**
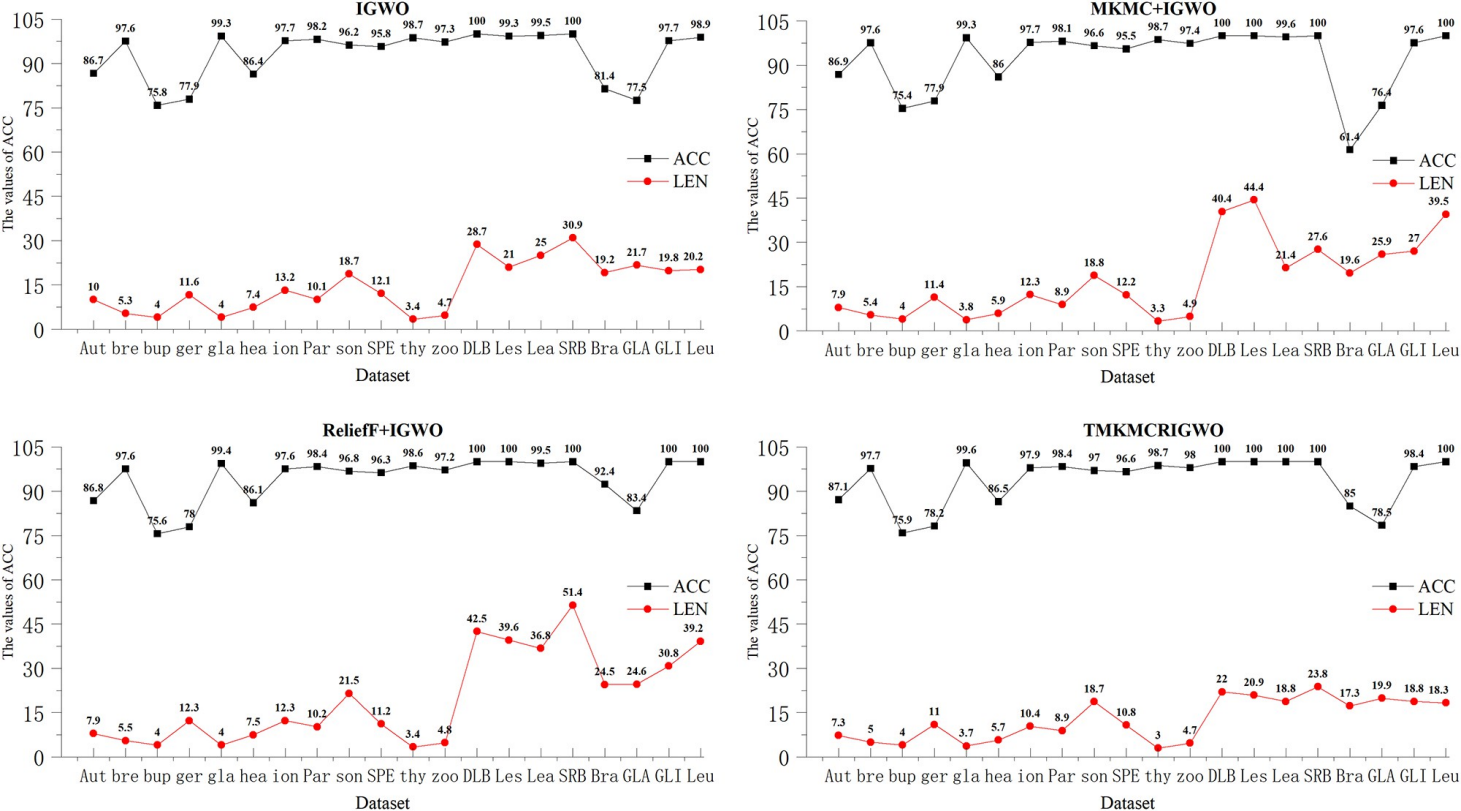
ACC and LEN for the second set of experimental algorithms. This figure shows the ACC and LEN of the second set of experimental algorithms.

In Fig 16, we compared the ACC and LEN of the four algorithms for the second set of experiments. On these three datasets, the higher average classification accuracy of the ReliefF+IGWO algorithm is due to the ability of the ReliefF algorithm to effectively identify important features within a certain period of time. On this basis, when combined with the wrapper algorithm, the advantages of the ReliefF algorithm and the wrapper algorithm can be fully utilized, and then the important features can be effectively retained. By combining the advantages of the two, the final result of improving the performance of the classifier and the prediction accuracy can be achieved. However, even though the ACC obtained by this algorithm is high, the LEN of the proposed algorithm is still the shortest length among these four algorithms.

**Fig 17:**
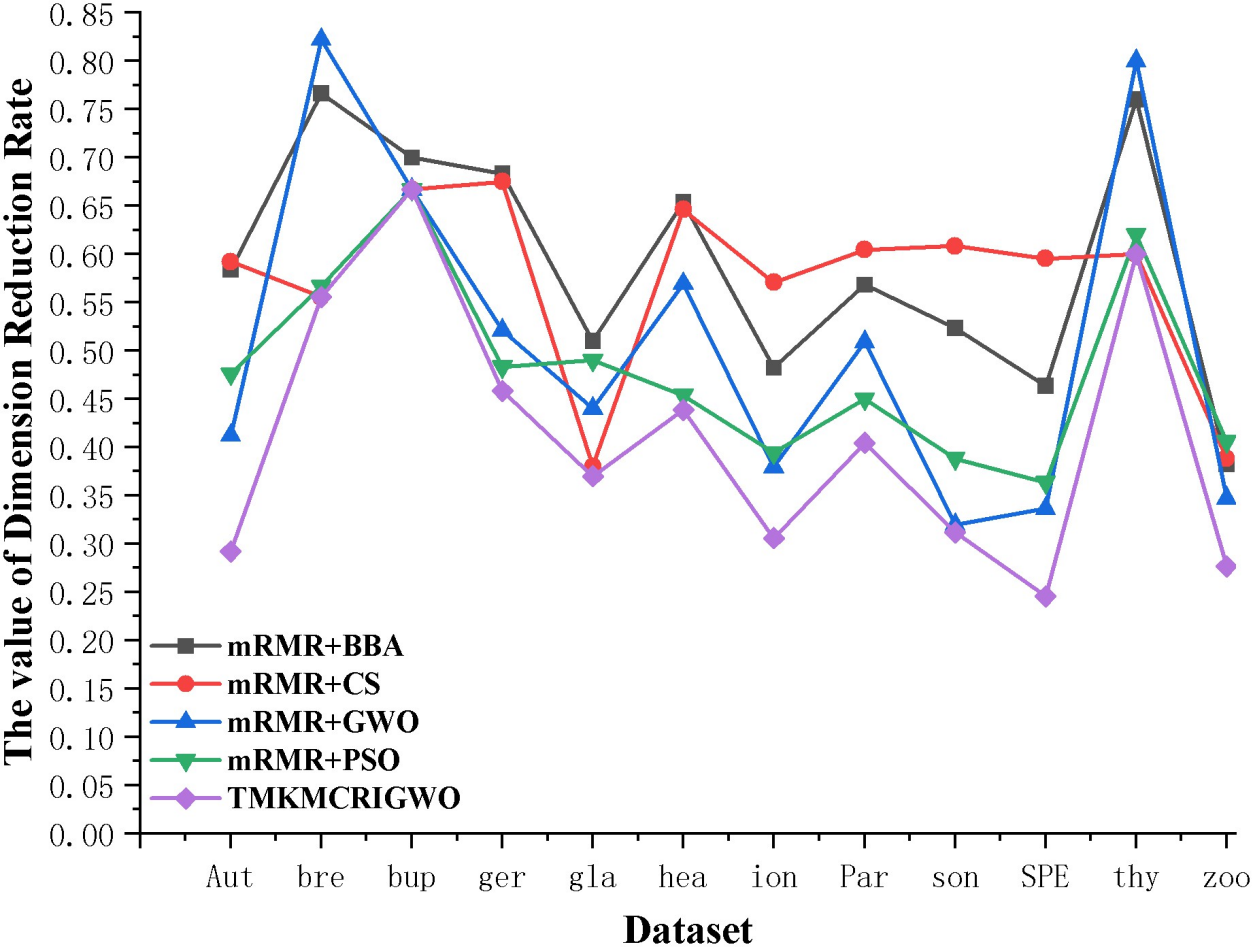
Dimension reduction rate of the third set of experimental algorithms on low-dimensional dataset. This figure shows the third set of experiments algorithm in 12 low-dimensional datasets on dimension reduction rate.

**Fig 18:**
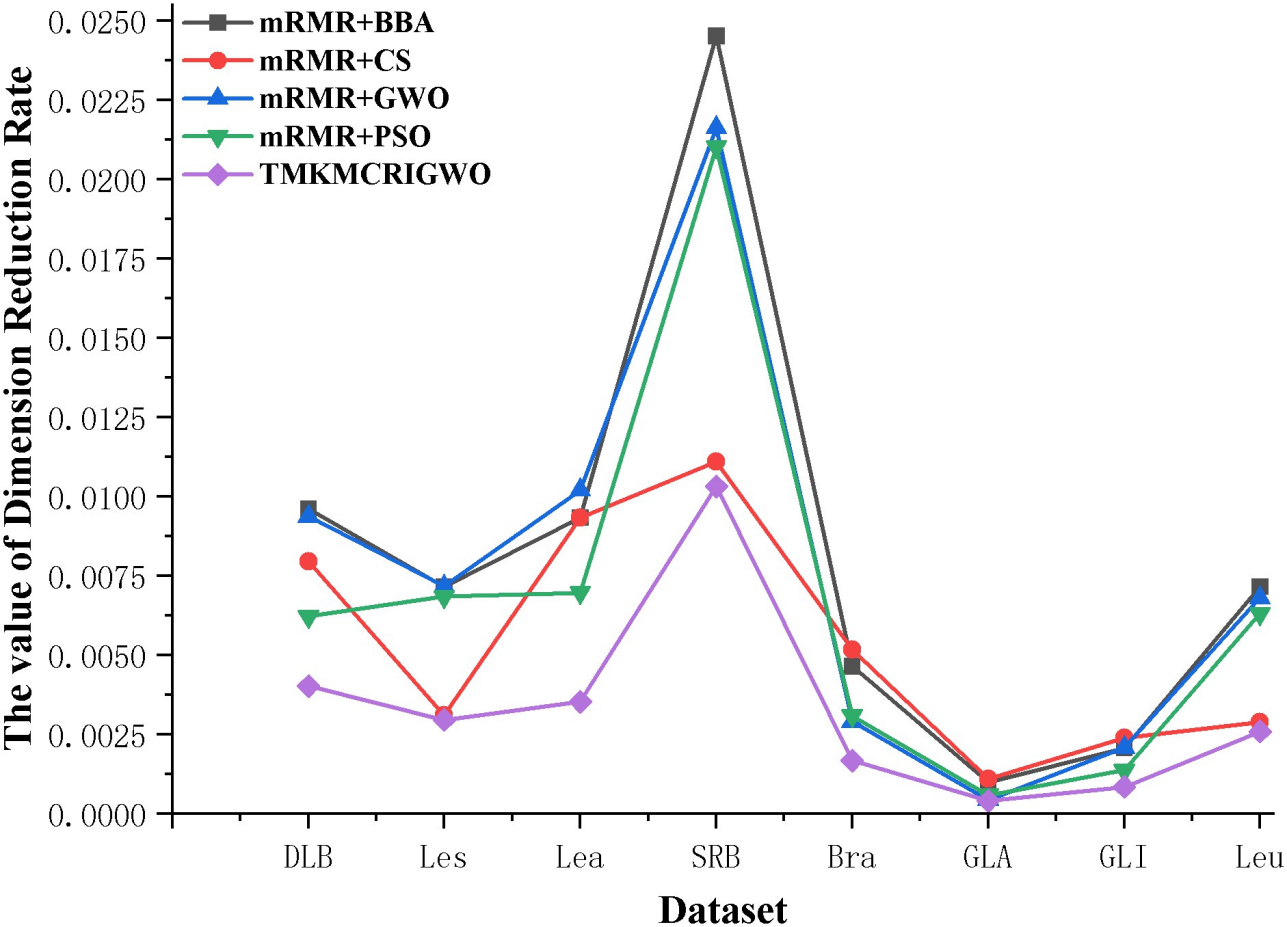
Dimension reduction rate of the third set of experimental algorithms on high-dimensional datasets. This figure shows the third set of experiments algorithm in 8 high-dimensional datasets on dimension reduction rate.

In Figs 17 and 18, we compared the dimension reduction rate of the third set of experiments again. It is observed that the dimension reduction rate of the TMKMCRIGWO algorithm is the lowest compared to the other hybrid algorithms in all the datasets. In low-dimensional datasets, the dimension reduction rates of both the TMKMCRIGWO algorithm and the other algorithms are above 25%. However, in high-dimensional datasets, the dimension reduction rates of these algorithms are basically in the range of 0.04% to 1%. Although the dimensionality reduction effect is more obvious in large datasets, the dimensionality reduction effect of the TMKMCRIGWO algorithm on low-dimensional datasets are all better than other algorithms. It also directly proves that the TMKMCRIGWO algorithm can better handle the dimensionality reduction of data and achieve more powerful effect.

**Fig 19:**
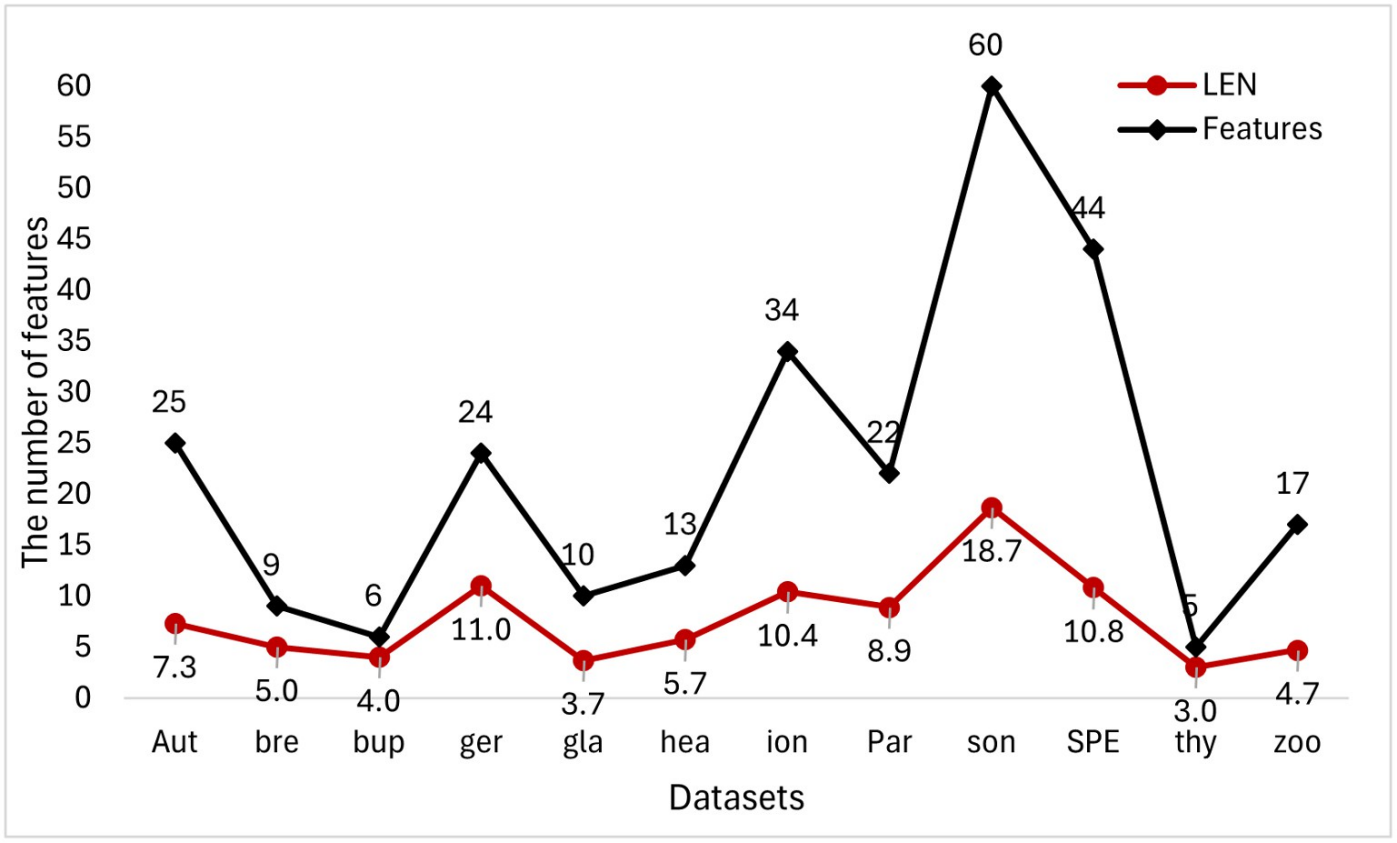
Degree of dimensionality reduction for 12 low-dimensional datasets. This figure shows the dimensionality reduction of the third set of experimental algorithms on 12 low-dimensional datasets.

**Fig 20:**
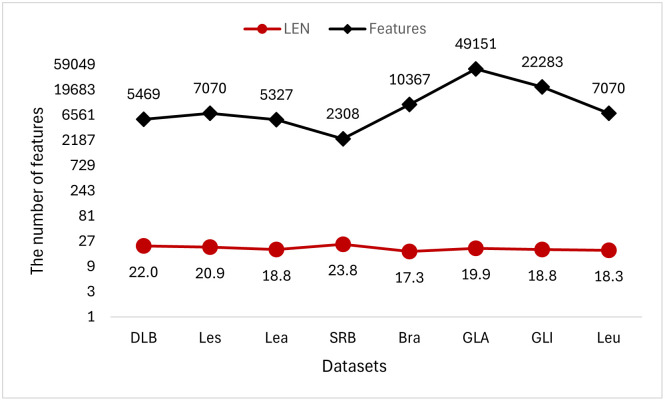
Degree of dimensionality reduction for 8 high-dimensional datasets. This figure shows the dimensionality reduction of the third set of experimental algorithms on 8 high-dimensional datasets.

**Fig 21:**
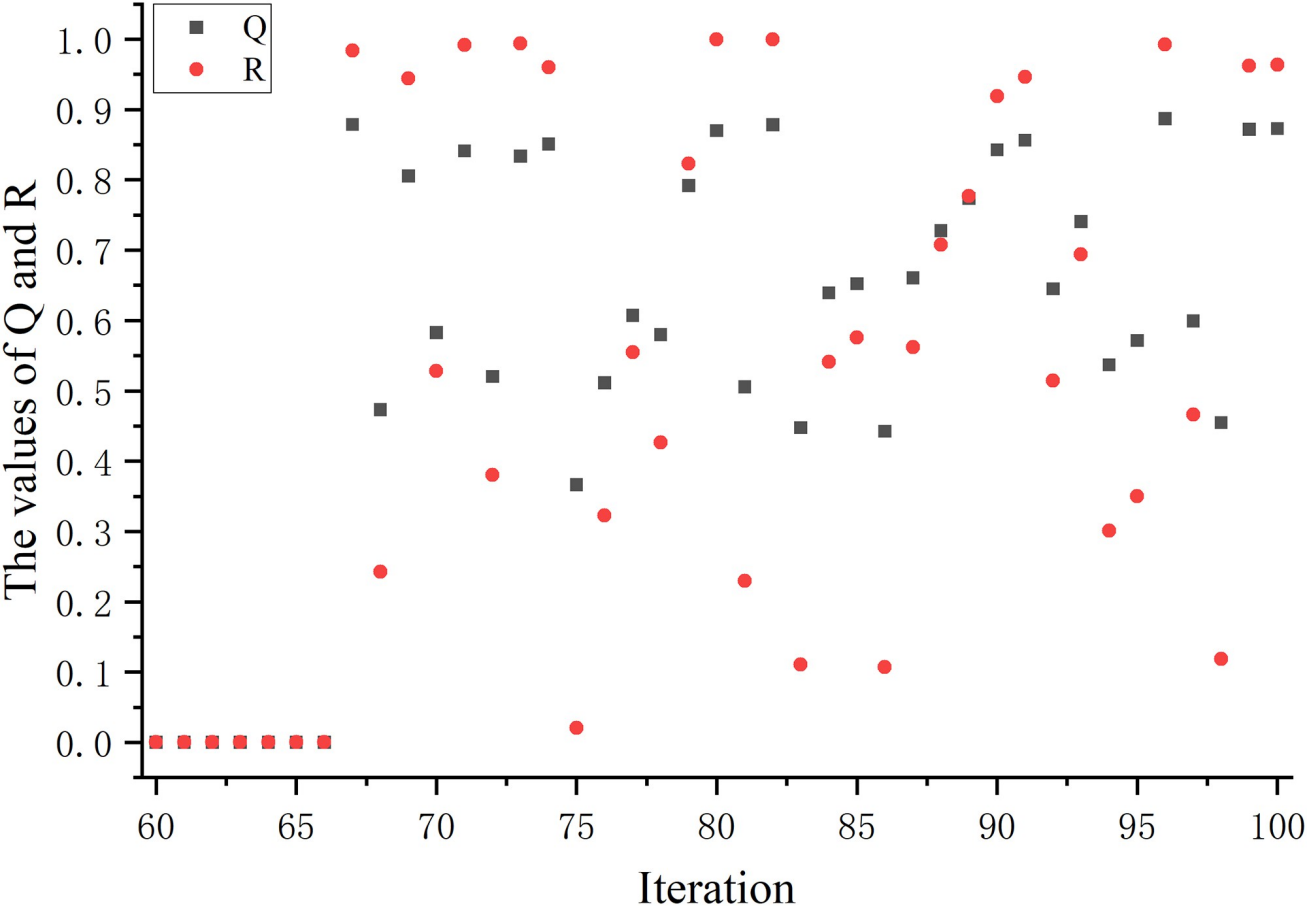
Variation of *Q* and *R* on the glass dataset. This figure shows the changes in *Q* and *R* on the low-dimensional dataset glass.

Figs 19–21 show the effect of low-dimensional dataset and high-dimensional dataset after dimensionality reduction by the TMKMCRIGWO algorithm, respectively. We plotted the original features of the dataset and the length of the subset of features after dimensionality reduction as line graphs. It can be clearly seen that our algorithm can reduce the dimensionality of the data both on the low-dimensional dataset and the high-dimensional dataset. It also shows that our algorithm is applicable on both low dimensional datasets and high dimensional datasets.

#### 2) Variation of the random disturbance factor

The effect of the random disturbance factor (*Q*) is to help the GWO explore more extensively in the search space, thus improving the ability to find an optimum. It increases the diversity of the algorithm and helps to avoid getting stuck in a local optimum without being able to find the global optimum. Thus, by introducing a *Q*, the algorithm is more probable to find a better solution. In this paper, the variation of the *Q* is determined by the mean and standard value of the variation with the position of the grey wolf.

To make the random disturbance factor flexible, we add a random number to it so that it can keep changing to avoid chance results. And the random disturbance factor is to adjust the change of position. Therefore, the change trend of the random disturbance factor directly affects the adjustment strategy of the position. Because of the large number of datasets used in this paper, we choose a representative dataset from the low-dimensional and the high-dimensional dataset respectively.

**Fig 22:**
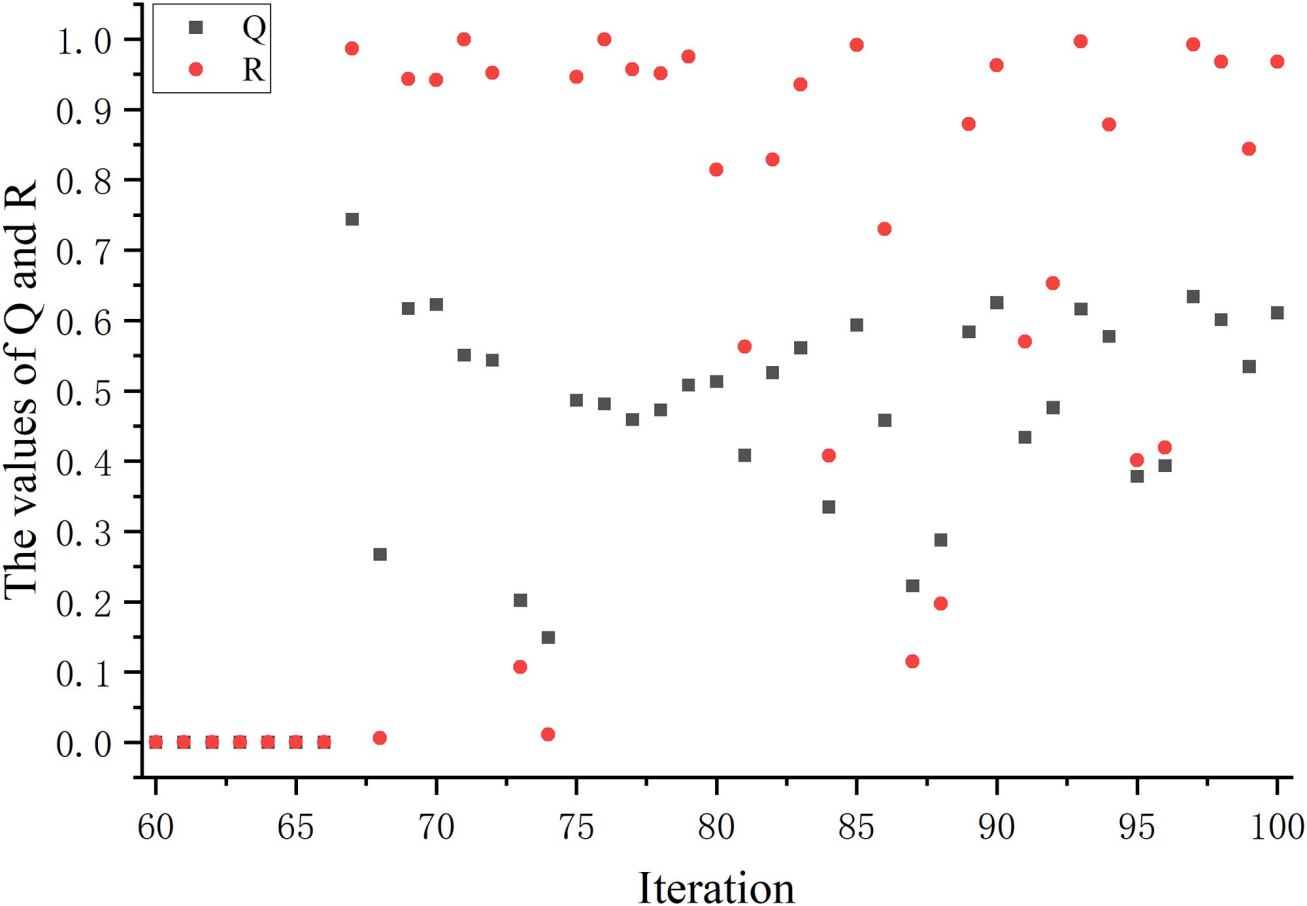
Variation of *Q* and *R* on the DLBCL dataset. This figure shows the changes in *Q* and *R* on the high-dimensional dataset DLBCL.

Figs 21 and 22 show the variation of the random disturbance factor under the influence of the random number *R* on the glass and DLBCL datasets, respectively. Since the random disturbance factor is regulated in the range after *T*_2/3_ time, the variations we see in *Q* and *R* are similarly in this range. On the glass dataset, the values of *Q* and *R* are closer in most cases. On the DLBCL dataset, the distribution of *Q* values tends to trend essentially on top of the *R* values, and the difference is large. It also shows that the random disturbance factor we introduced is follower, which can make the position change of the grey wolf rich in diversity.

#### 3) Variation of fitness values

In traditional algorithms, the fitness function only considers the average classification accuracy may ignore the effect of the feature subset length on the model performance, leading to the selection of an overly complex feature subset. An overly complex feature subset may increase the computational complexity of the model, reduce the model’s generalization ability, and even lead to overfitting problems.

Adopting a weight to measure the percentage of the ACC and LEN can regulate the trade-off between the two more flexibly. By choosing the weights reasonably, the performance and complexity of the algorithm can be balanced according to specific application scenarios and requirements. For example, if more attention is paid to the simplicity and generalization ability of the model, higher weights can be given to the LEN; if more attention is paid to the accuracy of the model, higher weights can be given to the ACC.

**Fig 23:**
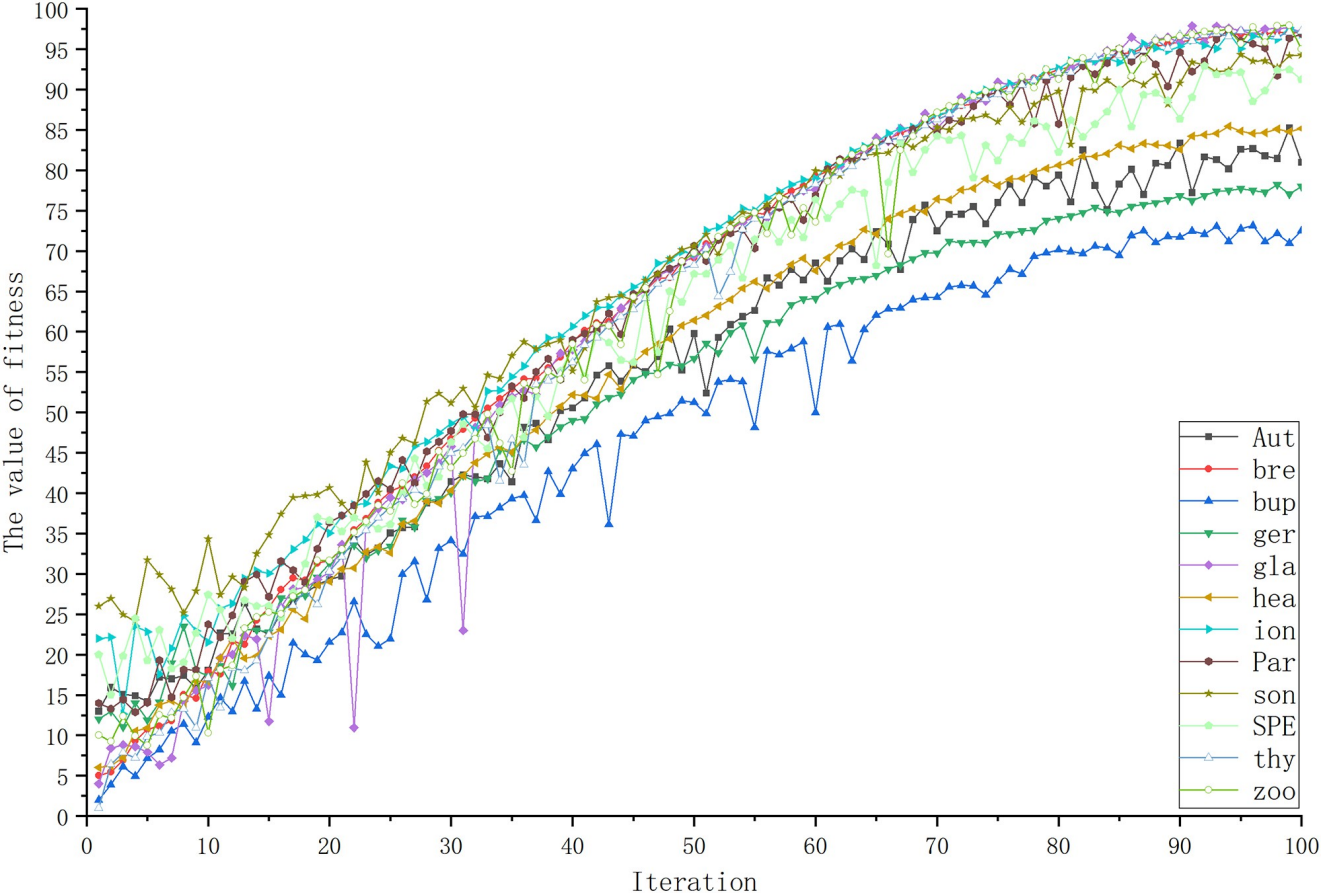
Trends of fitness values on low-dimensional datasets. This figure shows the trend of the fitness value over the number of iterations on the low-dimensional datasets.

**Fig 24:**
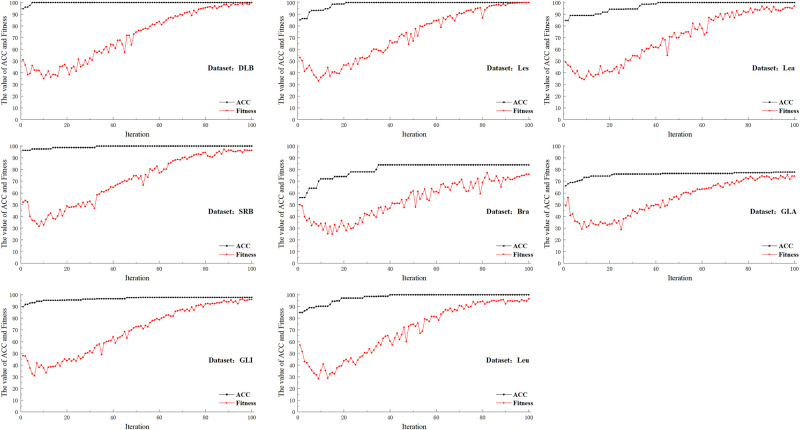
Trends of fitness values on high-dimensional datasets. The figure shows the trend of fitness values and ACC over the number of iterations on 8 high-dimensional datasets.

In this paper, we plotted the fitness values of the 20 datasets as they varied with the weighting parameter as a line graph. Because the weight values change with the number of iterations, the value of the fitness function also changes. As we can see from Figs 23 and 24, the value of the fitness function is not static or increasing (decreasing), it is regulated by the weight values according to the importance of ACC and LEN.

As shown in Figs 23 and 24, the change in the fitness value basically tends to increase during the iterative change. However, in high-dimensional datasets, because of the large amount of data, the iteration at the beginning may lead to a long feature subset length. Therefore, influenced by the weight values, the fitness value decreases by a small amount before increasing with the number of iterations. Through Fig 24, we can find this feature. In addition, from the figure we can still notice that the variation of the fitness value is strongly influenced by the ACC and is even close to the value of the average classification accuracy obtained. However, usually a higher fitness value indicates that the individual is closer to the optimal solution of the problem. It is also proved that the fitness value influences the performance of the individual in solving the problem, indicating that it is better capable of helping the algorithm to jump out of the local optimum. Therefore, adopting a weight value to measure the percentage of the ACC and LEN can regulate the trade-off relationship in the feature selection process more flexibly, thus better balancing the relationship between model performance and complexity. It also illustrates that the goodness of the fitness value indirectly determines the degree of superiority of the algorithm.

#### 4) Variation of the convergence factor

In traditional GWO, the convergence factor *a* is usually linearly decreasing during the iteration process from 2 to 0. It is designed to allow the wolves to gradually converge to the neighborhood of the optimal solution in the search space to increase the balance between global and local search. In this paper, we change the variation of the convergence factor *a* to vary randomly between [0, 2], which brings the following benefits.

1. Avoid premature convergence: the traditional linear decreasing approach may cause the algorithm to converge prematurely to a local optimum solution, whereas random variation can slow down this convergence and help to better explore the search space.

2. Increase the robustness of the algorithm: randomly varying the parameter *a* can make the algorithm more robust and better adaptable to different problems, helping to improve the algorithm’s global search capability.

**Fig 25:**
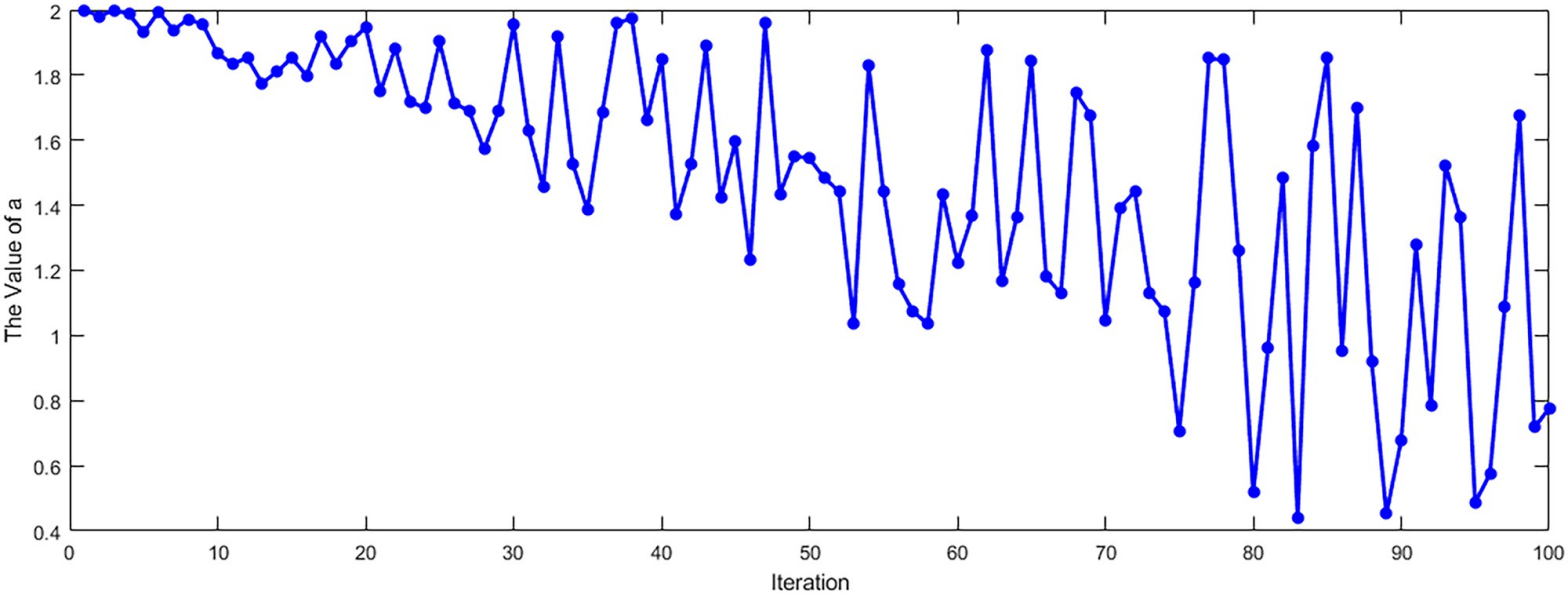
Trends of *a* on the Leukemia dataset. The figure shows the variation of the convergence factor *a* over the number of iterations on the Leukemia dataset.

Randomly varying the parameter *a* increases the diversity of the algorithm, allowing the wolves to have a greater ability to explore the search space and helping to avoid falling into local optima. Since the variation of the convergence factor is randomly varied, it is different in each dataset. Therefore, among the selected 20 datasets, we picked one of the most representative datasets to plot the image of convergence factor. Fig 25 shows the trend of *a* n the dataset Leukemia. It can be seen that *a* changes with the number of iterations, no longer decreasing in a single direction, but randomly changing suddenly high and low.

#### 5) Variation of the iteratively oscillatory random number *r*

In the traditional method, we calculate the grey wolf position after the corresponding position is not 0 or 1 value, according to a random number judgment attribute it to 0 or 1. However, it is not certain that the accuracy of the value and may even appear all 0 or all 1 of the results. To make the data more convincing and make the results more accurate, in this paper, the random number is changed to an iterative oscillatory selection method.

**Fig 26:**
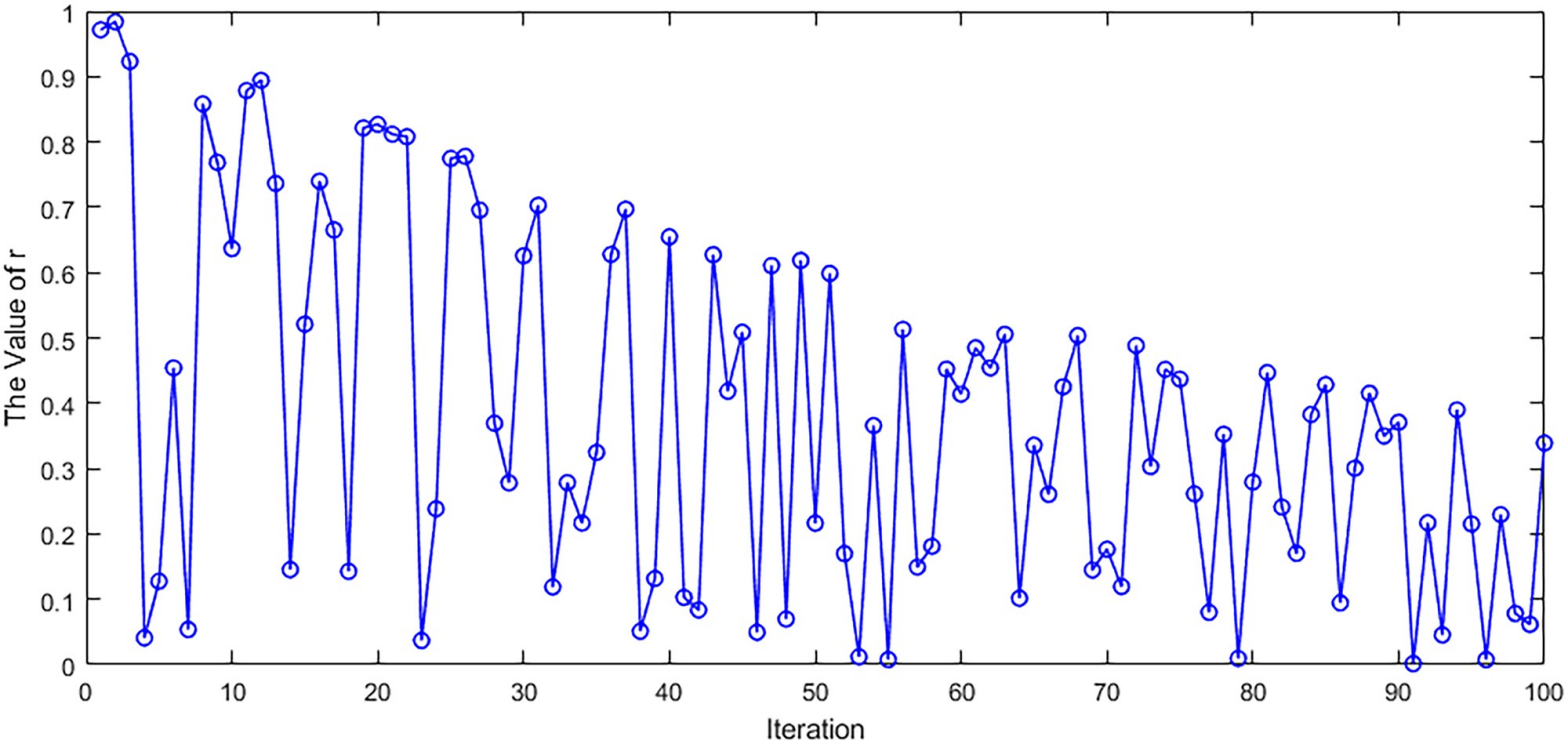
Trends of *r* on the automobile dataset. The figure shows the variation of *r* over the number of iterations on the Automobile dataset.

Since the selection of the grey wolf location is based on *r*, we selected the most representative Automobile dataset among all the datasets to present the trend of *r*. The *r* value of the Automobile dataset is not fixed at 0.5, but it varies with the number of iterations. As shown in Fig 26, the value of *r* is no longer fixed at 0.5, but varies with the number of iterations. Such an approach may make the selected subset richer in diversity.

The adjustment parameter is using the periodic sinusoidal vibrational function value that varies with the iteration coefficients. It can make the data have different distribution characteristics at different iteration numbers, thus increasing the diversity of the data. Meanwhile, the periodicity characteristics of this function can also make the data change regularly under different iteration times, which helps to better understand the change rule of the data. Therefore, changing the random number to a parameter that changes with the number of iterations can improve the credibility of the data and the accuracy of the results.

#### 6) Analysis of time complexity

Time complexity is an important concept in algorithm analysis, which is used to describe the increase in the execution time of an algorithm as the input size increases. Time complexity analysis can help us compare the performance of different algorithms and choose more efficient ones. In general, algorithms with lower time complexity are more efficient. In this paper, all the compared algorithms are compared with the proposed TMKMCRIGWO algorithm, and the time complexity is shown in Table 7.

**Table 7:** Time complexity.

No.	Algorithm	Time Complexity
1	MVO+SVM	*O*(*T* × *N* × *S*)
2	SA+SVM	*O*(*T* × *N* × *S*)
3	BBA+SVM	*O*(*T* × *N* × *S*)
4	GA+SVM	*O*(*T* × *N* × *S*)
5	ALO+SVM	*O*(*T* × *N* × *S*)
6	BDA+SVM	*O*(*T* × *N* × *S*)
7	CS+SVM	*O*(*T* × *N* × *S*)
8	GWO+SVM	*O*(*T* × *N* × *S*)
9	IGWO	*O*(*T* × *N* × *S*)
10	MKMC+IGWO	*O*(*T* × *N*^2^ × *S*)
11	ReliefF+IGWO	*O*(*T* × *N* × *M* × *S*)
12	mRMR+BBA	*O*(*T* × *N*^2^ × *S*)
13	mRMR+CS	*O*(*T* × *N*^2^ × *S*)
14	mRMR+GWO	*O*(*T* × *N*^2^ × *S*)
15	mRMR+PSO	*O*(*T* × *N*^2^ × *S*)
16	TMKMCRIGWO	*O*(*T* × (*K* × *N*) × *S*)

Where *T* denotes the total number of iterations, *K* denotes the number of features we selected, *N* denotes the number of features in the dataset, *M* denotes the number of nearest neighbors to be consideredand and *S* denotes the time required to execute the SVM classifier.

The time complexity of the wrapper algorithm is a little lower because there is no additional computation added to the wrapper algorithm. But in the hybrid algorithm, the filter algorithm is combined with the wrapper algorithm, so the time complexity of the hybrid algorithm is a little higher than the single wrapper algorithm. In ReliefF+IGWO, the time complexity of ReliefF algorithm requires judging the number of proximity obtained according to the number of features. The more features, the larger *M* is, conversely, the smaller it is.

In the TMKMCRIGWO algorithm proposed in this paper, two layers of filtering are first used to select a better subset of candidate features. If the original data set has 100 features, there may be 50 left after the first filter and 20 left after the second filter. Then, these 20 features are used as the original feature subset of the wrapper algorithm, which is more conducive to selecting the most relevant and minimum redundant features. As the number of features decreases, so does the computation time, so the time complexity decreases accordingly.

In Table 7, because the number of features we finally select is much less than the original number of features in the dataset, and *K* is much smaller than *N*, which means that *K* × *N* is less than *N*^2^. Therefore, this algorithm has lower time complexity than other hybrid algorithms. This also means that the TMKMCRIGWO algorithm can process more data or perform more iterations at the same time. This not only improves the performance but also the efficiency of the algorithm. In addition, the lower time complexity also means that the TMKMCRIGWO algorithm may be better suited for resource-limited situations, as it can accomplish computational tasks in a shorter period of time.

## 5. Conclusion

In feature selection process, hybrid algorithms can help to identify and eliminate redundant features in high or low dimensional data. Thus, in this paper, we propose a hybrid feature selection algorithm TMKMCRIGWO. The algorithm is formed by combining the bivariate filter algorithm MKMC in tandem with the univariate filter algorithm ReliefF, and then combining it with the improved IGWO wrapper algorithm. Through experiments, we verified that the algorithm obtains a higher average classification accuracy with lower length feature subsets. We can see that the hybrid algorithm shows better performance and results than a single algorithm in solving complex problems. By combining the advantages of different algorithms, the hybrid algorithm can overcome the limitations of a single algorithm and improve the accuracy and efficiency of problem solving.

Although the proposed algorithm has achieved certain results on some specific data sets and shown its advantages in solving specific problems, further optimization and improvement are still needed to realize the algorithm in a wider range of application scenarios. Future work may include extending the scope of application of the algorithm, optimizing the performance index of the algorithm, and carrying out the theoretical analysis and model improvement of the algorithm, etc. By continuing to promote these efforts, it is believed that the algorithm will be able to play an important role in more application areas and bring new breakthroughs and contributions to related research and practice.
